# The Variable Genomic NK Cell Receptor Locus Is a Key Determinant of CD4+ T Cell Responses During Viral Infection

**DOI:** 10.3389/fimmu.2020.00197

**Published:** 2020-02-20

**Authors:** Jana Raynor, Adora Lin, Sarah A. Hummel, Kristin Lampe, Michael Jordan, Kasper Hoebe, David A. Hildeman

**Affiliations:** ^1^Division of Immunobiology, Department of Pediatrics, Cincinnati Children's Hospital Medical Center, University of Cincinnati College of Medicine, Cincinnati, OH, United States; ^2^Immunology Graduate Program, Department of Pediatrics, Cincinnati Children's Hospital Medical Center, University of Cincinnati College of Medicine, Cincinnati, OH, United States

**Keywords:** NK cells, NK DC cross talk, CD4 T cells, viral infection, innate, adaptive immune response

## Abstract

Increasing evidence points to a key role for NK cells in controlling adaptive immune responses. In studies examining the role of CD1d on CD4+ T cell responses, we found that a line of CD1d-deficient mice on the C57BL/6J background had a homozygous 129 locus on chromosome 6 containing the entire NK cell gene cluster. Mice possessing this locus (C57BL/6.NKC^129^) displayed a >10-fold reduction in antigen-specific CD4+ T cell responses after intracranial infection with lymphocytic choriomeningitis virus (LCMV). Neither parental strain displayed defects in viral-specific CD4+ T cell responses. Interestingly, following infection, increased numbers of NK cells accumulated in the lymph nodes of C57BL/6.NKC^129^ mice and displayed enhanced *in vivo* functionality. Moreover, depletion of NK cells with anti-asialo-GM-1 antibody in C57BL/6.NKC^129^ mice resulted in a >20-fold increase in viral-specific CD4+ T cell responses. Mechanistically, we found that dendritic cell antigen presentation and early type I IFN production were significantly decreased in C57BL/6.NKC^129^ mice, but were restored in perforin-deficient C57BL/6.NKC^129^ mice or following NK depletion. Together, these data reveal that the variable genomic regions containing the activating/inhibitory NK cell receptors are key determinants of antigen-specific CD4+ T cell responses, controlling type I IFN production and the antigen-presenting capacity of dendritic cells.

## Introduction

Cytotoxic immune cells are critical for the clearance of altered-self, such as tumor cells and pathogen-infected cells. Cells involved in both innate and adaptive immunity have cytotoxic function, including natural killer T (NKT), natural killer (NK), and CD8+ T cells. In some viral infections, NKT and NK cells control viral replication early after infection (1–4), while a later-developing CD8+ T cell response is critical for elimination of most virally infected cells. Interestingly, there is mounting evidence showing that early NKT and NK cell responses can influence downstream T cell responses (5).

NKT cells are defined as an innate T cell lineage that express CD3 and αβ T cell receptor (TCR), as well as NK cell markers, such as NK1.1 (6). In mice, NKT cells display a limited TCR repertoire, with most expressing a rearrangement of the Vα14 and Jα18 segments paired with Vβ2, 7, or 8.2 chains (6, 7). These Vα14-Jα18 restricted NKT cells are referred to as invariant NKT (4). NKT cells recognize glycolipid or phospholipid antigens in the context of the MHC class I-like molecule CD1d, which consists of β_2_ microglobulin and the CD1d heavy chain (8). In addition, NKT cells have the capacity to rapidly secrete cytokines (e.g., IFN-γ, IL-2, and IL-4) and release granzymes and perforin for target cell lysis in a fashion similar to NK cells and cytotoxic CD8+ T cells (9–12). NKT cells have also been shown to contribute to the regulation of immune responses in a wide variety of disease states, including infection and autoimmunity (4, 6).

NK cells have an essential role in innate immunity, recognizing and eliminating target cells that present as infected or transformed cells. This is exemplified in humans deficient in NK cells and NK cell activity, who often suffer from persistent and life-threatening viral infections (13, 14). Along with cytokines, NK cell lytic function is critical for the clearance of certain pathogens, such as MCMV. However, more recently, NK cells have been recognized to play a key immunoregulatory role, controlling the onset and magnitude of adaptive immune responses. For instance, NK cells can negatively regulate anti-viral T cell responses directly by killing T cells (5, 15–18), or indirectly by limiting virus replication and APC function (19, 20). On the other hand, NK cell-mediated killing of target cells promotes cross-presentation and activation of CD8+ T cell responses (21). However, the mechanisms regulating NK cell activation and the extent to which NK cells impact adaptive immunity remain poorly understood.

NK cells do not express T cell receptors and are identified based on their cell surface expression of NKp46, CD49b (Dx5), and NK1.1 molecules (22). NK cells also express multiple surface receptors that have activating or inhibitory function, and the balance of these signals determines whether an NK cell will lyse an encountered target cell (22). These receptors include NKG2D, CD94/NKG2, the Nkpr1 gene family (*Klrb1*), and the Ly49 gene family (*Klra*), which are all encoded in a region on chromosome 6 termed the NK gene complex (NKC) (23, 24). The Ly49 receptors, which are functional homologs to the killer Ig-related receptors (KIRs) in humans (24), are either activating or inhibitory (23). Inhibitory Ly49/KIR receptors recognize MHC-I molecules and are critical in preventing NK cells from attacking healthy self (22). Interestingly, NKC genetic variation results in remarkable diversity, especially the Ly49 receptors, among different mouse strains such as C57BL/6 and 129 mice (24). To understand whether the NKC variance affects NK cell activity among inbred mouse strains, one study looked at NK functionality in mice congenic for the NKC region by inserting the 129 genomic NKC region into the C57BL/6 background (25). This study showed that NK cells from these congenic mice have a normal functional capacity to kill non-specific target cells compared to NK cells from wild-type C57BL/6 mice, and exhibit an enhanced rejection of missing-self targets (25). The NKC locus contains several genes that regulate NK functionality, beyond the Ly49 gene cluster, including CD94/NKG2A, NKG2D, and the Nkrp1 gene family. However, the impact of this locus on adaptive immunity remains unclear.

Here, we studied the role of NKT and NK cells in a mouse model of central nervous system (CNS) infection with lymphocytic choriomeningitis virus (LCMV). Intracranial LCMV infection induces a robust CD8+ T cell response, which is responsible for viral clearance and lethal immunopathological disease (26, 27). In mice deficient in CD8+ T cells, CD4+ T cells are able to mediate immunopathology but are not able to clear virus (28–32). During our initial studies examining the role of NKT cells on CD4+ T cell responses, we found that a particular line of CD1d-deficient mice had a 129 genetic locus on chromosome 6 that encompassed the entire NKC. These 129 congenic mice on a C57BL/6 background (C57BL/6.NKC^129^) had approximately a ninety percent decrease in LCMV-specific CD4+ T cell responses, which was associated with their reduced type I IFN production and the capacity of their dendritic cells to induce CD4+ T cell proliferation. Further, antibody-mediated depletion of NK cells or genetic deletion of perforin (Perforin-KO mice) in NKC^129^ congenic mice rescued DC functionality and the LCMV-specific CD4+ T cell response. Together, these data suggest that the NKC region is a key determinant of NK cell immunoregulatory function that controls the magnitude of virus-specific CD4+ T cell responses.

## Materials and Methods

### Virus

Mice were infected with the Armstrong-3 strain of LCMV, which was kindly provided by Dr. Rafi Ahmed (Emory University, Atlanta, GA). Virus was grown in BHK-21 cells (American Type Culture Collection, Manassas, VA), and viral titers of supernatants were determined by plaque assay on Vero cells as previously described (32).

### Mice and Viral Infections

C57BL/6 mice were purchased from Taconic (Hudson, NY). The two different strains of CD1d-deficient mice (CD1d-KO) were kind gifts from Dr. Luc Van Kaer (11), and Dr. Albert Bendelac (33, 34). Jα18-KO mice are deficient in invariant NKT cells (35). Perforin-deficient mice (Pfp-KO), 129X1/SvJ, and 129S1/SvImJ mice were purchased from The Jackson Laboratory (Bar Harbor, ME).

-KO mice were bred to C57BL/6.NKC^129^ and CD1d-KO.NKC^129^ mice in-house. C57BL/6.NKC^129^ congenic mice were generated by backcrossing CD1d-KO.NKC^129^ congenic mice with C57BL/6 mice, and screened for *CD1d1* by PCR and NK1.1 expression by flow cytometry. In the experiment in **Figure 3B**, the BL/6.NKC^129^ mice were heterozygous for the Slp76^Ace^ mutation, which acts as a recessive allele and does not influence a “missing self” NK cell response (36).

All mice were used within 8–16 weeks of age and were housed and bred under specific pathogen-free conditions in the animal facility in the Cincinnati Children's Hospital Research Foundation. Experimental procedures were reviewed and approved by the institutional animal care and use committee (IACUC) at the Cincinnati Children's Hospital Research Foundation. For intracranial (i.c.) infections with LCMV, mice were anesthetized by i.p. injection of ketamine/xylazine (100 mg/ml ketamine + 20 mg/ml xylazine mixture in saline) and then injected i.c. with 1 × 10^3^ plaque-forming units (p.f.u.) LCMV-Armstrong 3 in 30 μl PBS using a tuberculin syringe. Mock-infected mice received i.c. injections of 30 μl PBS.

### *In vivo* CD8+ T and NK Cell Depletions

Mice were injected i.p. with 0.25 mg of anti-CD8 depleting antibody (clone 2.43) 2 days before and 2 days after viral infection. Clone 2.43 antibody was generated in-house by either *in vivo* ascites or hybridoma production. For NK cell depletion, mice were injected i.p. with 20–30 μl of anti-asialo GM1 (Wako Chemicals USA) 2 days prior and 2 days after LCMV infection. >90% depletion of CD8+ T cells and NK cells was achieved.

### Flow Cytometry

Cervical lymph nodes (cLNs) or spleens were harvested and crushed through 100 μm filters (BD Falcon) to generate single-cell suspensions, and 1–2 × 10^6^ cells were stained with antibodies for flow cytometric analysis. For analysis of LCMV-specific T cells, MHC class II tetrameric staining reagents were generated as previously described (37, 38). The tetramer we employed detects T cells specific for LCMV glycoprotein amino acids 61–80, which is an immunodominant LCMV epitope (39, 40). For some experiments, we used an I-A^b^ gp66-77-strepdavidin-phycoerythrin-labeled tetramer from the NIH tetramer core facility (41, 42). No significant differences were observed in the detection of LCMV-specific T cell responses using homemade compared to NIH tetramers. Cells were additionally stained with anti-CD44, CD16/32, and CD4 antibodies (eBioscience or BD Biosciences, San Jose, CA).

For NK cell analysis, cells were stained with fluorescently labeled antibodies against NKp46, Dx5, NK1.1, and TCRβ antibodies (eBioscience or BD Biosciences, San Jose, CA). For DC analysis, cells were stained with fluorescently labeled antibodies against CD11b, MHC Class II, CD11c, CD8α, PDCA-1, B220, XCR1, SIRPα, Gr-1, Zbtb46 antibodies. Data were acquired on an LSRII flow cytometer (BD Biosciences) or a Canto-II and analyzed using CellQuest Pro or FACSDiva software (BD Biosciences) or FlowJo software.

### Genome-Wide Single Nucleotide Polymorphism Analysis

To assess the background of CD1d-KO mice we performed an initial genome wide SNP analysis using a SNP map containing 347 markers informative for C57BL/6J and 129X1/SvJ genetic backgrounds as described before (43). A total of 3–5 mice per group (high, medium, and low CD4^+^ T cell responses and NK1.1 expression) were genotyped using the Illumina GoldenGate Assay. Following the initial genome wide scan, the chromosome 6 region identified was further defined by assessing additional SNPs using PCR and the following primers:

rs3715240: Fwd CGCAAGCTCCATTCGAGACAT; Rvs CCAGGAGGAGCCAGGCCATAAT

rs3023092: Fwd CCTGCTAGCAAAGGCTCACTT; Rvs GGCTACAGAGTCTCCTGTGCAA

rs3710061: Fwd CAGACCCACAGACTCACAGAT; Rvs CCAACAGGCCTATGCCTTCT

rs3663781: Fwd GGCCAGCAGAACAAACATTGA; Rvs GCCTGTCGGTGTGCAGTATG.

### Type I IFN Luciferase Assay

Serum levels of type I IFN were assessed using a L929-ISRE luciferase reporter assay as described before (21).

### *In vivo* Cytotoxicity Assay to Assess NK Cell Responses

The *in vivo* NK cell cytotoxicity assay was performed as previously described (36). Briefly, C57BL/6 mice or C57BL/6^Slp76Ace+/−^.NKC^129^ mice were injected intravenously with 3 × 10^6^ low CFSE-labeled “missing-self” target cells (β2M-deficient splenocytes), intermediate CFSE-labeled allogeneic target cells (Balb/c splenocytes), and high CFSE-labeled wild-type control C57BL/6 splenocytes. Further, WT recipient mice received NK depleting antibody (anti-asialo GM1) or isotype control. Blood was collected 24 h later, and the cells were assessed by flow cytometry. The percent killing was calculated from the ratio of target cells to control cells in NK-depleted recipients compared to isotype control recipients.

### *Ex vivo* CD4+ T Cell Proliferation Assay

Mice were infected i.c. with LCMV Armstrong 3, sacrificed on days 4, 5, or 6 post-infection, and the cLN were harvested. DCs were isolated from the cLN using a CD11c positive-selecting MACs column (Miltenyi Biotec, San Diego, CA). LCMV-specific CD4+ T cells were isolated from the spleens of naïve SMARTA TCR transgenic mice (39) and labeled with CFSE. Naïve SMARTA T cells and DC from LCMV infected mice were co-cultured for 72 h, and SMARTA CD4+ T cell proliferation was assessed by the decrease in CFSE expression via flow cytometry.

### Statistical Analysis

For most analyses a standard *t*-test was used when data appeared normally distributed. For some experiments where data points were approaching zero, a non-parametric Mann–Whitney *U*-test was used. Statistical analyses were performed using either Excel or GraphPad Prism.

## Results

### LCMV-Specific CD4+ T Cell Responses Are Reduced in CD1d-KO Mice

**Figure 1:**
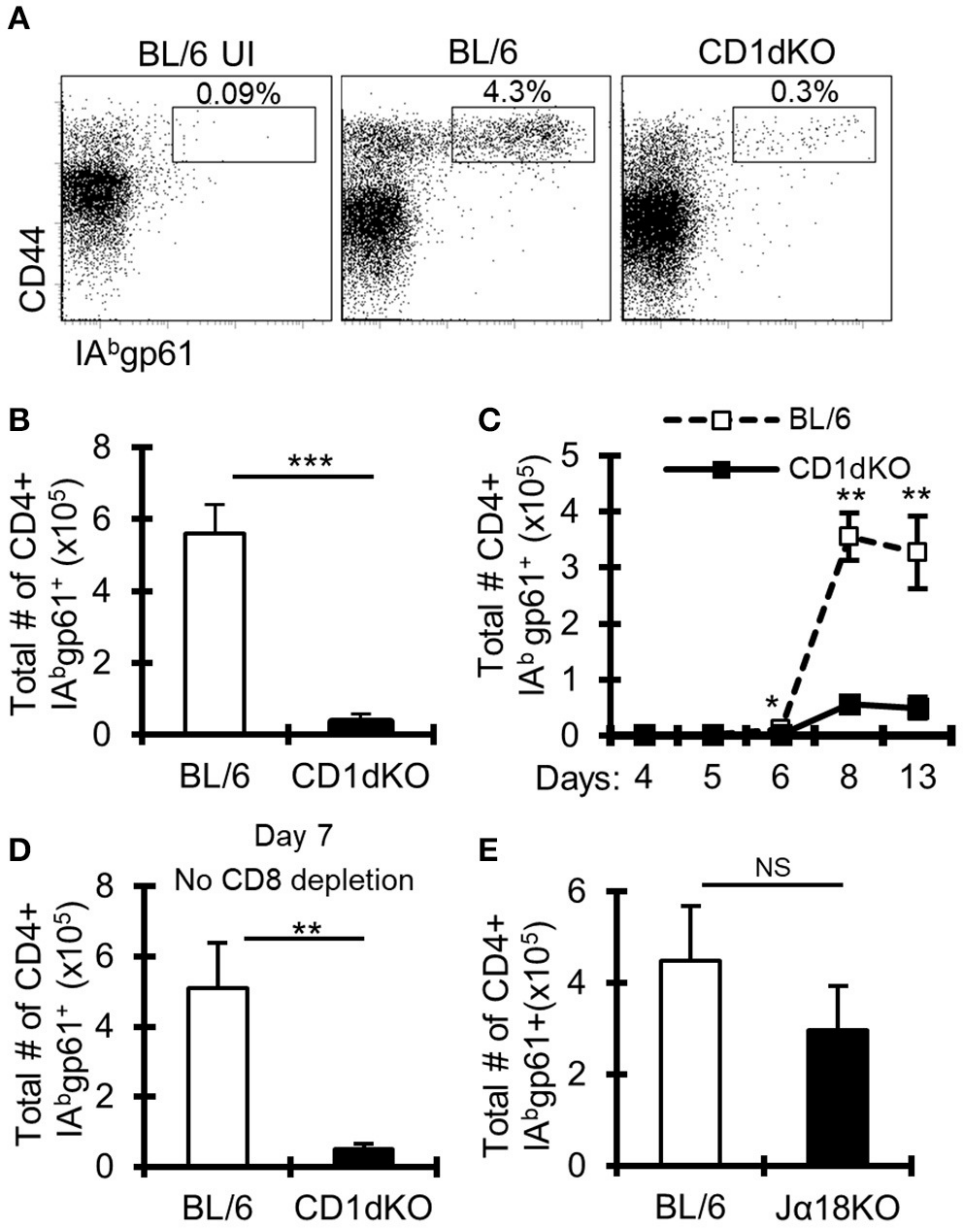
LCMV-specific CD4+ T cell responses are reduced in CD1d-KO mice. C57BL/6 and CD1d-KO mice (*n* = 3–5 mice/group) were infected i.c. with 1 × 10^3^ p.f.u. LCMV Armstrong 3. Unless otherwise indicated, all mice were depleted of CD8+ T cells. **(A,B)** LCMV-infected mice were sacrificed on day 8 post-infection, cervical lymph nodes (cLN) were harvested, single cells were stained for CD4, CD44, CD16/32, and IA^b^gp-61 tetramer, and analyzed by flow cytometry. **(A)** Dot plots show the frequency of CD4+ CD16/32- cells that are CD44hi gp61-tet+. **(B)** Data show the total number of cells that are CD4+ CD16/32- CD44hi gp-61tet+ (±SE). **(A,B)** Data are representative of more than three independent experiments. **(C)** Mice were sacrificed on days 4, 5, 6, 8, and 13 post-LCMV infection, and data show the total number of cells that are CD4+ CD16/32– CD44hi gp-61tet+ (±SE). **(D)** Mice were sacrificed on day 7 post-LCMV infection. Mice did not receive anti-CD8 depleting antibody. Data show the total number of cells that are CD4+ CD16/32– CD44hi gp-61tet+ (±SE). **(E)** C57BL/6 and Jα18KO mice (*n* = 3–4 mice/group) were sacrificed on day 8 post-LCMV infection. Data show the total number of cells that are CD4+ CD16/32– CD44hi gp-61tet+ (±SE). *p ≤ 0.05, **p ≤ 0.01, ***p ≤ 0.001 (Student's *t*-test).

Previous work suggested limited involvement of NKT cells in controlling the magnitude of anti-LCMV T cell responses (44, 45); however, these studies involved peripheral and not central nervous system (CNS) viral infection. As prior work implicated NKT cells in immune responses in the CNS (46, 47), we determined the role of NKT cells in CNS LCMV infections. To do this, we infected CD1d deficient mice (CD1d-KO), which lack NKT cells, intracranially (i.c.) with LCMV. To avoid the mortality associated with i.c. LCMV infection, mice were additionally depleted of CD8+ T cells. On day 8 after infection, the frequency and number of IA^b^-gp61-specific (gp61-sp.) CD4+ T cells in the draining cervical lymph nodes (cLNs) failed to increase in CD1d-KO mice compared to wild-type (WT) C57BL/6 mice (Figures 1A,B). Similar results were obtained when LCMV-specific CD4+ T cells were analyzed in the brain on day 8 (Supplemental Figures 1A,B), and there was a minor, but significant decrease in the total number, but not the frequency, of LCMV-specific CD4+ T cells in the spleen after i.p. LCMV infection (Supplemental Figures 1C–E). Further, the loss in gp61-sp. CD4+ T cells was not due to a delayed response, as CD1d-KO mice still had significantly reduced gp61-sp. CD4+ T cells at day 13 post-infection (Figure 1C). As we used an antibody against CD8α to deplete CD8+ T cells, it was possible that this drove the loss of CD8α+ dendritic cells, which in turn reduced the CD4+ T cell response. To test this, mice were i.c. LCMV infected without receiving anti-CD8α depleting antibody and sacrificed on day 7, prior to the development of fatal choriomeningitis. Importantly, CD1d-KO mice still had a significant loss in gp61-sp. CD4+ T cells without CD8α+ cell depletion (Figure 1D). Thus, the decreased LCMV-specific CD4+ T cell response was not due to the effects of CD8α-depleting antibody.

Two major subsets of NKT cells have been identified, so-called invariant and non-invariant NKT cells. Invariant NKT cells are characterized by their expression of a conserved Vα14-Jα18 TCRα chain (4), while non-invariant NKT cells have a more diverse TCRα chain (6). To determine whether the loss of gp61-sp. CD4+ T cells in CD1d-KO mice is due to the loss of invariant or non-invariant NKT cells, Jα18 deficient (Jα18-KO) mice, which lack invariant NKT cells (35), were infected. Interestingly, Jα18-KO mice had normal CD4+ T cell responses to i.c. LCMV infection, showing that invariant NKT cells are not required for anti-LCMV CD4+ T cell responses (Figure 1E). These data suggest a role for non-invariant NKT cells in promoting gp61-sp. CD4+ T cell responses during LCMV CNS infection.

### LCMV-Specific CD4+ T Cell Responses Vary Among Different CD1d-KO Mouse Lines

**Figure 2:**
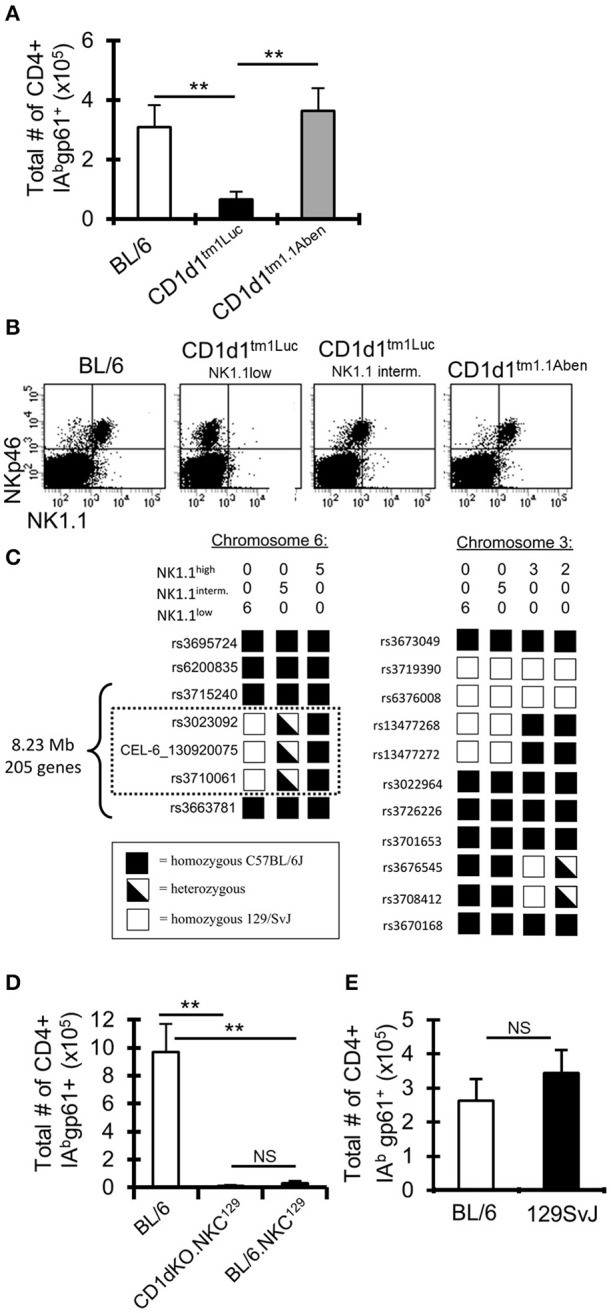
A 129 locus on chromosome 6 in C57BL/6 mice impairs the anti-LCMV CD4+ T cell response. **(A)** C57BL/6 and two different lines of CD1d-KO mice (CD1d1^tm1Luc^ and CD1d1^tm1.1Aben^) (*n* = 3–5 mice/group) were infected i.c. with 1 × 10^3^ p.f.u. LCMV Armstrong 3. The mice were sacrificed on day 8 post-infection, cervical lymph nodes (cLN) were harvested, single cells were stained for CD4, CD44, CD16/32, and IA^b^gp-61 tetramer, and analyzed by flow cytometry. Data show the total number of cells that are CD4+ CD16/32– CD44hi IA^b^gp-61tet+ (±SE). **p ≤ 0.01, (Student's *t*-test). **(B)** Dot plots show the expression of NKp46 and NK1.1 on cLN cells that are TCRβ-. **(C)** SNP analysis was done on CD1d1^tm1.1Aben^ mice that were NK1.1hi (*n* = 5) and CD1d1^tm1Luc^ mice that were either NK1.1int (*n* = 5) or NK1.1low (*n* = 6). **(D,E)** C57BL/6, CD1d-KO.NKC^129^, BL/6.NKC^129^, and 129SvJ mice (*n* = 3–5 mice/group) were sacrificed on day 8 post-LCMV infection. Data show the total number of cells that are CD4+ CD16/32– CD44hi IA^b^gp-61tet+ (±SE). **p ≤ 0.05, (Mann–Whitney test) and are representative of three independent experiments with similar results.

Our CD1d-KO mice originated from Luc Van Kaer's laboratory (“CD1d1^tm1Luc^”) (11). To validate our results obtained using the CD1d1^tm1Luc^ mice, we used an independent CD1d-KO mouse line generated by Albert Bendelac's laboratory (“CD1d1^tm1.1Aben^”) (33, 34). Surprisingly, following i.c. LCMV infection, the CD1d1^tm1.1Aben^ mice had normal gp61-sp. CD4+ T cell numbers compared to WT C57BL/6 mice, while CD1d1^tm1Luc^ had reduced gp61-sp. CD4+ T cells (Figure 2A). Both mouse lines were confirmed to lack CD1d protein expression (Supplemental Figure 2). These data demonstrate that the decrease in the gp61-sp. CD4+ T cell response in CD1d-KO mice is independent of NKT cells.

### Single Nucleotide Polymorphism (SNP) Analysis Reveals a 129 Locus on Chromosome 6 in CD1d-KO Mice

As CD1d and NKT cells did not appear to contribute to the altered LCMV-sp. CD4+ T cell response in CD1d-KO mice, we next examined markers expressed by NK cells as NK cells have been reported to control CD4+ T cell responses (15, 17). Interestingly, in our colony of CD1d1^tm1Luc^ mice we observed, in most mice, that TCRβ-NKp46+ cells had low or intermediate expression levels of NK1.1, while TCRβ-NKp46+ cells in C57BL/6 and CD1d1^tm1.1Aben^ mice all had high expression of NK1.1 (Figure 2B). Importantly, the low expression of NK1.1 correlated with the impaired LCMV-specific CD4+ T cell response (Figures 2A,B). It is known that the anti-NK1.1 antibody (clone PK136) binds to NK1.1 on certain backgrounds, such as C57BL/6, but not on other backgrounds, such as BALB/c and 129S1, due to a mutation that affects the epitope binding site for the PK136 antibody (48). This observation led us to question if perhaps the CD1d1^tm1Luc^ mice had a mutation in the NK1.1 gene or possessed a NK1.1 allele from one of these other genetic backgrounds.

Given that both CD1d-KO lines were generated in 129 ES cells and then backcrossed onto the C57BL/6 background (11, 33, 34), a genome-wide single nucleotide polymorphism analysis was performed on these mice to determine how much of the 129 genome remained. Both the CD1d1^tm1Luc^ and CD1d1^tm1.1Aben^ mice were found to be similarly backcrossed to the C57BL/6J background (~97% pure C57BL/6J, Supplemental Table 1). Interestingly, besides the 129 genomic DNA surrounding the CD1d locus, the CD1d1^tm1Luc^ mice contained a small 8.23 Mb region on chromosome 6 (encoding 205 genes, Supplemental Table 2), that was either homozygous or heterozygous for 129 (Figure 2C) and that correlated with the low or intermediate expression of NK1.1, respectively. Interestingly, this region on chromosome 6 includes the NK gene complex (NKC), which contains many genes that contribute to NK cell licensing and function (23, 24). The CD1d1^tm1Luc^ mice that were homozygous for 129 on chromosome 6 we have termed CD1d-KO.NKC^129^. This analysis also explains the results for the differential NK1.1 staining in Figure 2B as these mice were homozygous at the NKC.129 locus, while the mice that were intermediate for NK1.1 staining were heterozygous for the NKC.129 locus.

### Having the Chromosome 6 129 NK Locus on the C57BL/6 Background Drastically Impairs CD4+ T Cell Responses to LCMV

To determine whether the impaired LCMV-specific CD4+ T cell response in CD1d-KO.NKC^129^ mice relied on either the CD1d locus or the NKC^129^ locus, CD1d-KO.NKC^129^ mice were backcrossed to C57BL/6 mice to generate congenic BL/6.NKC^129^ mice. BL/6.NKC^129^ mice express CD1d and are homozygous for the 129 locus on chromosome 6. After i.c. LCMV infection, the BL/6.NKC^129^ mice have reduced gp61-sp. CD4+ T cells, similar to CD1d-KO.NKC^129^ mice (Figure 2D). Additionally, WT 129 mice have normal gp61-sp. CD4+ T cell numbers compared to WT C57BL/6 mice (Figure 2E). These data show that the 8.3 Mb region of the NKC^129^ locus existing within the C57BL/6 background limits the CD4+ T cell response to LCMV.

### NK Cells Inhibit LCMV-Specific CD4+ T Cell Responses in Both CD1d-KO.NKC^129^ and BL/6.NKC^129^ Mice

**Figure 3:**
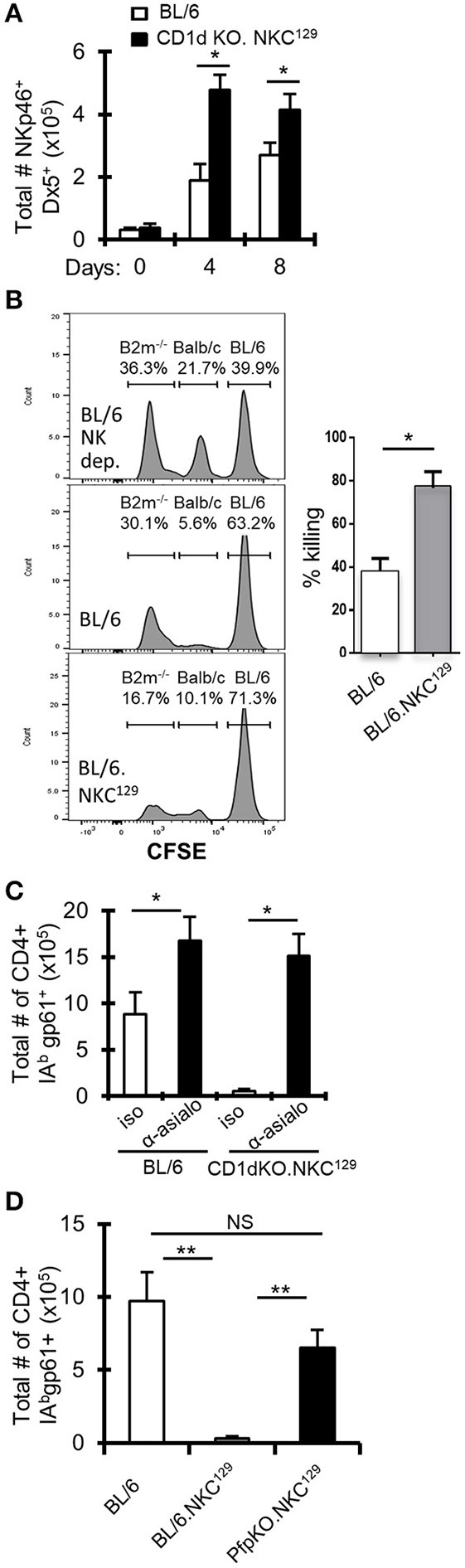
NK cells inhibit LCMV-specific CD4+ T cell responses in NKC^129^ congenic mice. **(A)** C57BL/6 and CD1d-KO.NKC^129^ mice (*n* = 3 mice/group) were infected i.c. with 1 × 10^3^ p.f.u. LCMV Armstrong 3, and cLN cells were analyzed on days 0, 4, and 8 post-infection. Data show the total number of TCRβ- NKp46+ Dx5+ cells (±SE). *p ≤ 0.05 (Student's *t*-test). **(B)** *In vivo* cytotoxicity assay assessing rejection of β2M-deficient splenocytes in C57BL/6J and BL/6^Slp76+/−^.NKC^129^ mice. The percentage killing was determined after 24 h by comparing the level of CFSE labeled non-targets (C57BL/6J) and target cells (β2M^−/−^) in C57BL/6J vs. BL/6.NKC^129^ *p ≤ 0.05 (Student's *t*-test). **(C)** C57BL/6 and CD1d-KO.NKC^129^ mice (*n* = 3–4 mice/group) received anti-asialo GM1 or isotype control antibody and were sacrificed on day 8 post-LCMV infection. Data show the total number of cells that are CD4+ CD16/32– CD44hi IA^b^gp-61tet+ (±SE). *p ≤ 0.05 (Student's *t*-test). **(D)** C57BL/6, BL/6.NKC^129^, and CD1dKOxPfp^−/−^.NKC^129^ mice (*n* = 3 mice/group) were sacrificed on day 8 post-LCMV infection. Data show the total number of cells that are CD4+ CD16/32– CD44hi IA^b^gp-61tet+ (±SE) and are representative of at least three independent experiments. **p ≤ 0.05 (Mann–Whitney test).

The 8.3 Mb NKC locus on chromosome 6 contains many genes that are critical for NK education/licensing and activation, including the highly polymorphic *Klra* (Ly49) gene family that has significant variation between the C57BL/6 and 129 mouse strains (24). Indeed, other studies have shown that possession of the 129 genome at the *Klra* gene complex in C57BL/6 mice affects NK functionality (25). Further, it has been shown that NK cells can limit the T cell response to LCMV infection [reviewed in (5)]. Interestingly, CD1d-KO.NKC^129^ (CD1d1^tm1Luc^) mice have a significant increase in NK cell numbers during LCMV infection (Figure 3A), which was also observed in BL/6.NKC^129^ mice, but not in CD1d1^tm1.1Aben^ mice, whether assessed by NKp46 staining or Dx5 staining (Supplemental Figure 3A). This increase in NK cells appeared to be related to infection because, at baseline, there were minimal differences in the frequency or numbers of NK cells as well as in naïve and effector/effector memory CD4+ T cells or their state of activation at baseline as assessed by CD69 expression (Supplemental Figures 3B,C, 4A–C).

To determine whether NK cell function was altered in BL/6.NKC^129^ mice, we assessed their ability to eliminate β2M-deficient splenocytes using an *in vivo* cytotoxicity assay (36). Importantly, compared to WT C57BL/6 mice, BL/6.NKC^129^ mice exhibited an increased rejection of missing-self targets, but no difference in their ability to kill allogeneic targets (Figure 3B). We also did not detect a difference in degranulation (assessed by CD107α expression) or production of IFN-γ after stimulation with either IL-12p70+IL-18 or PMA+ Ionomycin (Supplemental Figure 4D). We next tested whether the increased NK cells and/or their altered activity contributed to control of antiviral CD4+ T cell responses in CD1d-KO.NKC^129^ mice by depleting NK cells with anti-asialo-GM1 antibody. Depletion of NK cells resulted in a near 20-fold increase in the numbers of gp61-sp. CD4+ T cells in CD1d-KO.NKC^129^ mice and even resulted in a nearly two-fold increase in WT mice (Figure 3C). To determine whether the cytotoxic function of NK cells contributed to the control of LCMV-sp. CD4+ T cell responses, we generated perforin-deficient BL/6.NKC^129^ (CD1dKOxPfp^−/−^.NKC^129^) mice. Strikingly, the absence of perforin restored the numbers of gp61-sp. CD4+ cell after i.c. LCMV infection (Figure 3D). Thus, NK cells and perforin limit the LCMV-specific CD4+ T cell response in CD1d-KO.NKC^129^ and BL/6.NKC^129^ mice.

### DC Antigen-Presentation Is Impaired in NKC^129^ Congenic Mice and Restored in the Absence of Perforin

**Figure 4:**
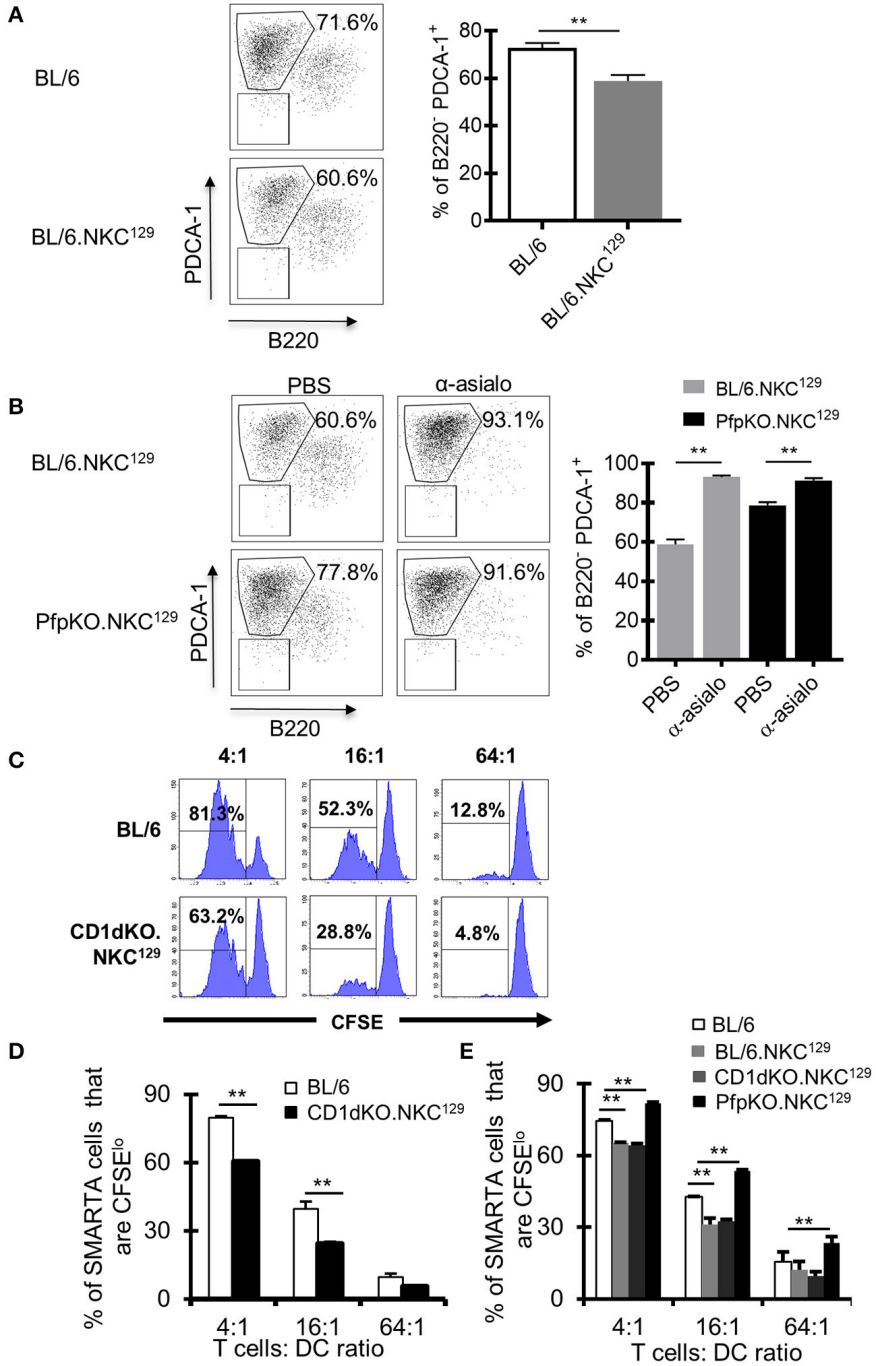
Dendritic cell antigen-presentation is impaired in C57BL/6 mice with the NKC^129^ locus and is restored in the absence of perforin. **(A,B)** Groups of C57BL/6 and BL/6.NKC^129^ mice (*n* = 3–4 mice/group) were infected i.c. with 1 × 10^3^ p.f.u. LCMV Armstrong 3 and were injected with either PBS or anti-asialo-GM1 (days −2, 2) and sacrificed on day 4 after infection. Single cell suspensions from cLN were generated, and cells were stained with antibodies to characterize dendritic cells (DCs). Graphs show **(A)** the frequency of PDCA-1+ cells (gated from CD11b+CD11c+) on day 4 after infection, and **(B)** after NK cell depletion. **(C,D)** C57BL/6 and CD1d-KO.NKC^129^ mice (*n* = 3–5 mice/group) were infected i.c. with 1 × 10^3^ p.f.u. LCMV Armstrong 3. CD11c+ cells were isolated from the cLN on day 6 post-LCMV infection and co-cultured with CFSE labeled naïve SMARTA CD4+ T cells for 72 h. Data show **(C)** histograms and **(D)** graph of the frequency of SMARTA CD4+ T cells that are CFSE^lo^ that were cultured with DCs from either C57BL/6 or CD1d-KO.NKC^129^ mice (±SE). Data are representative of 2 independent experiments with similar results. **(E)** C57BL/6, BL/6.NKC^129^, CD1d-KO.NKC^129^, and CD1dKOxPfp^−/−^.NKC^129^ mice (*n* = 3–5 mice/group) were infected i.c. with 1 × 10^3^ p.f.u. LCMV Armstrong 3. CD11c+ cells were isolated from the cLN on day 6 post-LCMV infection and co-cultured with CFSE labeled naïve SMARTA CD4+ T cells for 72 h. Data show the frequency of SMARTA CD4+ T cells that are CFSE^lo^ (±SE) across multiple T cell:DC ratios. **p ≤ 0.01 (Student's *t*-test).

NK cells can inhibit antiviral CD4+ T cell responses directly by killing activated CD4+ T cells (17), potentially through NKG2A recognition of Qa-1 expressed on activated CD4+ T cells (15), or indirectly by limiting the capacity of APCs to stimulate T cells (19, 20). In our model of LCMV CNS infection, it was unclear whether NK cells were directly or indirectly impairing the LCMV-specific CD4+ T cell response. First, we determined if having the NKC^129^ locus in the C57BL/6 background affected the ability to control virus. We found no difference in viral load in purified lymph node dendritic cells nor in whole brain tissue between C57BL/6 and BL/6.NKC^129^ mice (Supplemental Figures 5A,B). Next, we found that NK cells from CD1d-KO.NKC^129^ mice did not display an enhanced ability to kill CD4+ T cells *in vitro* relative to C57BL/6 mice (Supplemental Figure 5C). We next considered that NK cells might be killing DCs. Indeed, the frequency of CD11c+ CD11b+ PDCA-1+ DCs were decreased in BL/6.NKC^129^ mice compared to C57BL/6 controls (Figure 4A). We also analyzed cDC1 (CD11c+, MHC class II+, CD8α+, XCR1+, SIRPα-), cDC2 (CD11c+, CD11b+, MHC class II+, SIRPα+) and migratory DCs (CD11c+, CD8+, XCR1+) on days 2 and 4 after infection, the only difference we found was a slight, but significant, decrease in the numbers of mDCs in BL/6.NKC^129^ mice on day 2 after infection (Supplemental Figure 6). In addition, depletion of NK cells with anti-asialo-GM1 significantly enhanced the frequency of PDCA1+ DCs in BL/6.NKC^129^ mice, but to only a minor extent in CD1dKOxPfp^−/−^.NKC^129^ (Figure 4B). These data suggest that the killing of DCs might underlie the defective CD4+ T cell responses in BL/6.NKC^129^ mice.

To directly test this possibility, we employed an assay that we had used previously to show a minor fraction of DCs promote antigen presentation during LCMV infection (49). Next, we took CD11c+ DCs from LCMV-infected BL/6.NKC^129^ or CD1d-KO.NKC^129^ mice and tested their ability to induce *ex vivo* proliferation of gp61-sp. SMARTA CD4+ TCR Tg cells. Consistent with the reduction of gp61-sp. CD4+ T cell responses in LCMV-infected BL/6.NKC^129^ and CD1d-KO.NKC^129^ mice, DCs from these mice also had a significantly impaired ability to stimulate SMARTA T cells relative to BL/6 WT controls (Figures 4C,D). Across multiple T cell to DC ratios, SMARTA T cell proliferation was reduced with DCs from CD1d-KO.NKC^129^ mice (Figures 4C,D). Next, we tested whether the reduced ability to present antigen required perforin. Similar to the results in restoring CD4+ T cell responses, we found that the decrease in antigen-presentation was rescued by the deletion of perforin in CD1dKOxPfp^−/−^.NKC^129^ mice (Figure 4E) Together these data are consistent with a scenario in which NK cells kill DCs and limit their ability to prime CD4+ T cell responses.

### Type I Interferon Production Is Reduced in CD1dKO.NKC^129^ Mice and Partially Restored With NK Depletion

**Figure 5:**
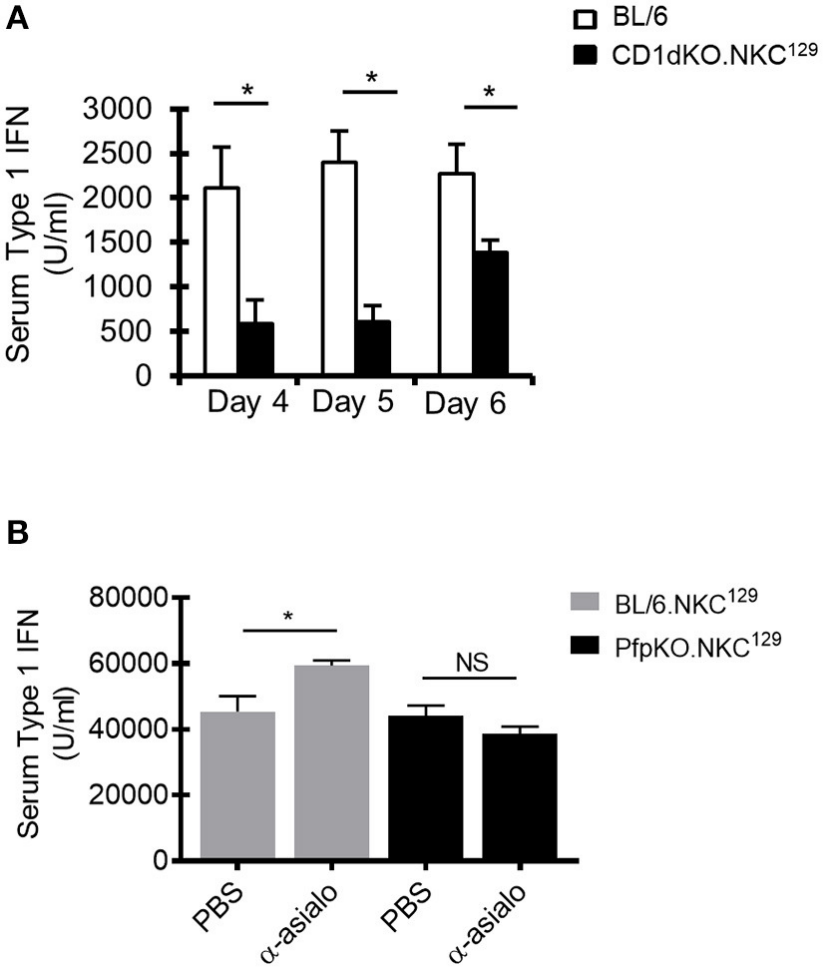
NK cells limit type I interferon production. **(A)** C57BL/6 and BL/6.NKC^129^ mice (*n* = 3–5 mice/group) were infected i.c. with 1 × 10^3^ p.f.u. LCMV Armstrong 3. Serum was collected on days 4, 5, or 6 post-infection. Data show the average type I IFN in the serum, as determined by an L929-ISRE luciferase reporter assay (±SE). **(B)** BL/6.NKC^129^ and CD1dKOxPfp^−/−^.NKC^129^ mice (*n* = 3–5 mice/group) were infected i.c. with 1 × 10^3^ p.f.u. LCMV Armstrong 3 and received either anti-asialo GM1 or PBS (d-2, 2). Serum was collected on day 4 post-LCMV infection and assessed for type I IFN as described in section Materials And Methods. Data are representative of at least 3-independent experiments. *p ≤ 0.05 (Student's *t*-test).

As DCs are a major source of type I IFN after LCMV infection, and our data suggested an impairment of DC function in CD1dKO.NKC^129^ mice, we examined serum levels of type I IFN in infected C57BL/6 WT and CD1d-KO.NKC^129^ mice. Consistent with their decreased DC function *ex vivo*, serum type I IFN levels were significantly reduced at several points after LCMV infection (Figure 5A). Further, the depletion of NK cells partly restored type I IFN levels in BL/6.NKC^129^ mice, but not in CD1dKOxPfp^−/−^.NKC^129^ mice (Figure 5B). Together, these data suggest that NK cells in NKC^129^ congenic mice may limit CD4+ T cell responses by impairing the functionality of dendritic cells both at the level of antigen-presentation and cytokine production.

## Discussion

The NK gene complex (NKC) is evolutionarily diverse and has high allelic polymorphism among different mouse strains (24). This genetic variation has been utilized to better understand the regulation of NK cell homeostasis and function by generating chimera mice at the NKC locus (25). Such studies have demonstrated that NK functionality is altered when the 129 genome at the NKC locus is inserted in the C57BL/6 background (25, 36). Here, using an independent BL/6.NKC^129^ congenic mouse line, we showed that NK cells from BL/6.NKC^129^ mice have increased function relative to C57BL/6 mice during LCMV infection and have linked this increased function to negative regulation of anti-viral CD4+ T cell responses. Importantly, we found that this enhanced NK function resulted in impaired type I IFN production, reduced antigen-presentation by DCs, and a dramatic loss in downstream LCMV-specific CD4+ T cell responses.

The mechanism regulating altered NK functionality in our NKC^129^ congenic mice remains unclear; however, the comparable CD4+ T cell responses between WT C57BL/6 and 129 mice suggest that a receptor/ligand interaction between the C57BL/6 and 129 genetic backgrounds influences the NK response to LCMV infection. There are many potential receptors responsible for the altered NK functionality in NKC^129^ mice because the NKC contains the majority of genes encoding activating and inhibitory NK receptors, including CD94/NKG2A, NKG2D, the Nkrp1 gene family, and the Ly49 gene family (22–24). Our data suggests that CD94/NKG2A and NKG2D do not play a dominant role in regulating NK cells during LCMV infection, as we did not observe a significant loss in CD4+ T cells or DCs that express their cognate ligands, Qa-1 (CD94/NKG2A) or H60, Rae-1, or Mult-1 (NKG2D), nor an enrichment of cells expressing these markers in the absence of perforin-mediated killing. Interestingly, the 129S1 and C57BL/6 strains have variable *Ly49* haplotypes with 129S1 mice possessing 19 Ly49 genes, while C57BL/6J mice encode only 15 *Ly49* genes (24).

These Ly49 receptors, which include activating and inhibitory receptors, recognize MHC-I. Ly49 receptors play a critical role in NK cell recognition of altered or missing-self cells, as well as NK cell “education” during development (22). Indeed, prior work showed that Ly49 recognition of self-MHC-I serves to “educate” NK cells during development, called “licensing,” and thus enhance their responsiveness in the periphery (5, 50, 51). However, during MCMV infection in C57BL/6 mice, “unlicensed” NK cells (Ly49C/I–, Ly49G2+) are more responsive and are better able to control virus compared to licensed NK cells (Ly49C/I+) (52). Another report showed that CD1d1^tm1Luc^ mice had reduced NK cell responses to missing self (53). However, these CD1d1^tm1Luc^ mice did not possess the same NK region from 129 mice as ours because they reported normal NK1.1 expression (53). Instead, our NKC^129^ mice have the Ly49 profile of 129S1 mice, similar to what was previously reported in an independent 129 congenic mouse at the NKC locus (25). Importantly, in this latter work, Patel et al. showed that their congenic mice exhibited increased recognition of MHC-I-deficient targets, consistent with our prior and present work in BL/6.NKC^129^ mice (36). Thus, while the contributions of specific Ly49 receptors in regulating NK responses during LCMV infection are still unknown, the altered Ly49 expression profile between the NKC^129^ and BL/6 mice may significantly affect NK responsiveness.

NK cells can affect T cell responses directly (5, 15–18) or indirectly by influencing DC function (19, 20). Direct mechanisms can be mediated by cytokines, such as IL-10 or IFNγ (54), or direct cytolytic activity predominantly through the perforin/granzyme mediated pathway. Our data are consistent with the killing function of NK cells playing a role as the absence of perforin restored CD4+ T cell responses in NKC^129^ mice. However, we failed to observe direct killing of activated CD4+ T cells by NK cells *in vitro*. Nonetheless, the potential direct and indirect regulation of CD4+ T cell responses in NKC^129^ mice is not necessarily mutually exclusive. Indeed, NK cells limited the type I IFN production in NKC^129^ mice, and type I IFN can promote T cell protection from NK-mediated killing by upregulating NK inhibitory ligands (55, 56). Thus, the influence of NK cells on anti-viral CD4+ T cell responses is likely mediated by multiple mechanisms.

Our data support a model of indirect regulation of CD4+ T cell responses by NK cells in NKC^129^ mice via the culling of DCs. Supporting this concept, we observe a loss in type I IFN production and a reduction in antigen-presenting capacity of DCs in an NK-perforin dependent manner. If NK cells were directly killing CD4+ T cells, we would not have expected an effect on systemic type I IFN responses as we observed. However, we cannot rule out an effect of NK cells directly killing CD4+ T cells. Our data is in agreement with other studies that have shown that NK cells can impair antigen presentation and stimulatory capacity of APCs during viral infections (19, 20). Importantly, these data may at least partly explain observed effects of NK cells on Tfh responses (57, 58). We also showed a decrease in CD11c+CD11b+PDCA1+ DCs on day 4 as well as mDCs on day 2 after infection between BL/6.NKC^129^ mice compared to C57BL/6 mice. Indeed, the reduced capacity to induce CD4+ T cell activation *in vitro* and decreased type I IFN production strongly suggest that the quality of DC responses were impaired in mice possessing the 129 NKC locus. It is likely that this impaired DC response was due to the loss (due to killing) of a small subset of DC poised for antigen presentation, rather than a decrease in quality of total DC. Indeed, we previously showed that a small population of CD11c+ DCs actually presents antigen to CD8+ T cells specific for LCMV (49). It is also likely that the reduced type I IFN, known to promote the T cell response (59, 60), also contributes to the significant loss in LCMV-specific CD4+ T cells in NKC^129^ congenic mice. We also cannot rule out a potential cell intrinsic defects in CD4+ T cells driven by their expression of genes within the NKC^129^ cluster in a C57BL/6 genetic background (note that CD4+ T cell responses are normal in 129 mice). Nonetheless, the sub-optimal DC capacity to induce CD4+ T cell activation; reduced type I IFN levels in NKC^129^ congenic mice; and the potential for NK cell mediated killing of CD4+ T cells, add up to an environment that is not conducive for optimal anti-viral CD4+ T cell responses.

Overall, our data reveal a surprising and key modulatory role for the genomic variable NK cell receptor regions in the control of virus-specific CD4+ T cell responses. The drastic changes in the magnitude of CD4+ T cell responses observed in the context of different congenic NKC regions warrants further investigation to identify the exact molecular and cellular mechanisms that mediate such control of virus-specific adaptive immune responses. Moreover, these changes pose an important question whether similar gene-gene interactions involving NK cell inhibitory or activating receptors define human T cell responses and, for instance, determine T cell responses during viral/bacterial infections or during vaccination and/or affect the development of auto-immune diseases.

## Data Availability Statement

All datasets generated for this study are included in the article/Supplementary Material.

## Ethics Statement

The animal study was reviewed and approved by Cincinnati Children's Hospital Institutional Animal Care and Use Committee.

## Author Contributions

JR, AL, SH, KL, and DH performed experiments. JR, AL, MJ, KH, and DH designed experiments. JR, AL, KH, and DH wrote the manuscript. All authors reviewed the manuscript.

### Conflict of Interest

The authors declare that the research was conducted in the absence of any commercial or financial relationships that could be construed as a potential conflict of interest.
