# Supplementary material for: The Variable Genomic NK Cell Receptor Locus Is a Key Determinant of CD4+ T Cell Responses During Viral Infection
Source: Front Immunol. 2020 Feb 20;11:197. doi: 10.3389/fimmu.2020.00197 (PMC7044186; doi:10.3389/fimmu.2020.00197)
Supplement: Supplementary file 1 [file Presentation_1.pptx]

## Slide 1
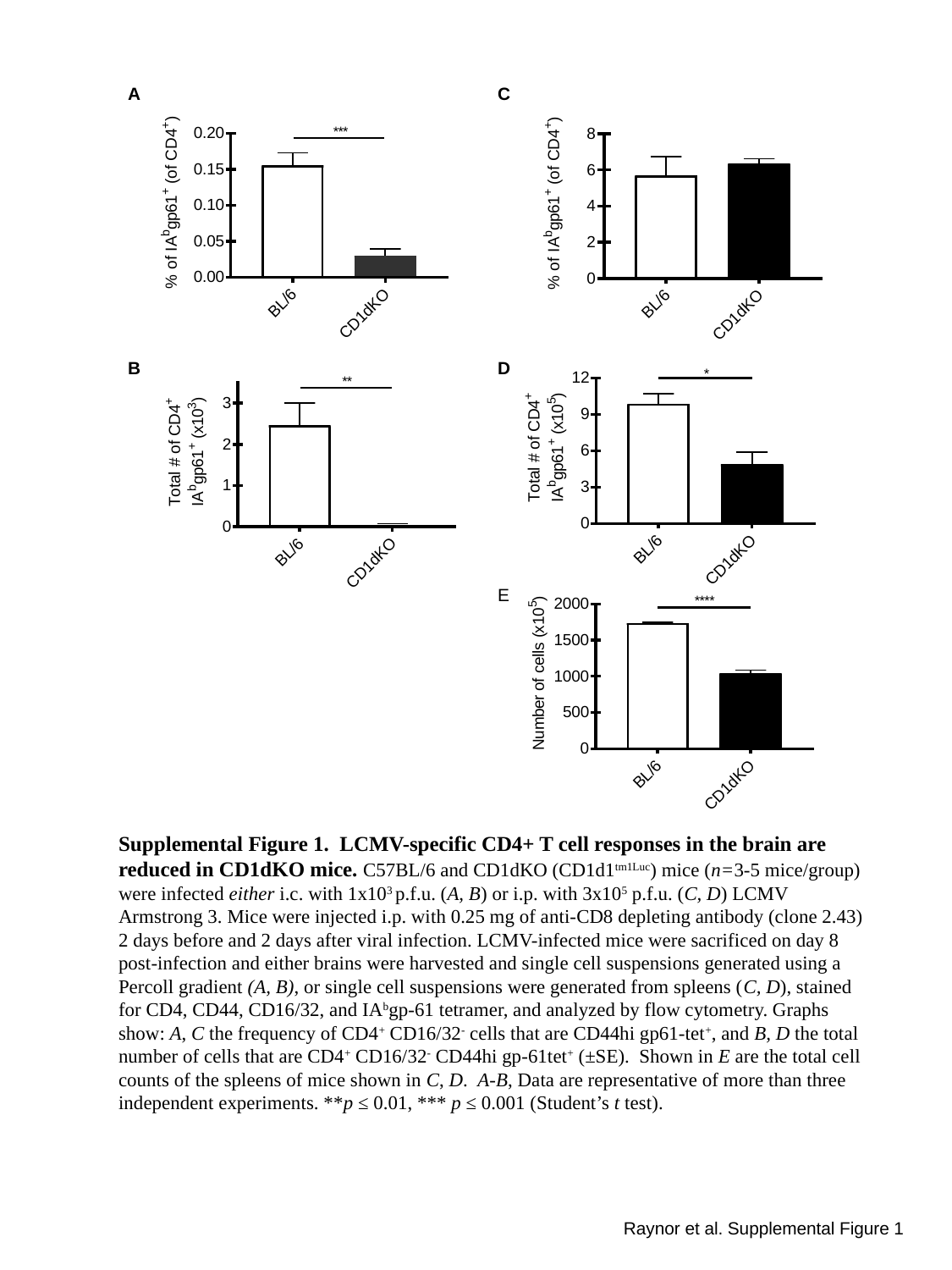

A
C
D
B
E
Supplemental Figure 1. LCMV-specific CD4+ T cell responses in the brain are reduced in CD1dKO mice. C57BL/6 and CD1dKO (CD1d1tm1Luc) mice (n=3-5 mice/group) were infected either i.c. with 1x10^3^ p.f.u. (A, B) or i.p. with 3x10^5^ p.f.u. (C, D) LCMV Armstrong 3. Mice were injected i.p. with 0.25 mg of anti-CD8 depleting antibody (clone 2.43) 2 days before and 2 days after viral infection. LCMV-infected mice were sacrificed on day 8 post-infection and either brains were harvested and single cell suspensions generated using a Percoll gradient (A, B), or single cell suspensions were generated from spleens (C, D), stained for CD4, CD44, CD16/32, and IAbgp-61 tetramer, and analyzed by flow cytometry. Graphs show: A, C the frequency of CD4+ CD16/32- cells that are CD44hi gp61-tet+, and B, D the total number of cells that are CD4+ CD16/32- CD44hi gp-61tet+ (±SE). Shown in E are the total cell counts of the spleens of mice shown in C, D. A-B, Data are representative of more than three independent experiments. **p ≤ 0.01, *** p ≤ 0.001 (Student’s t test).
Raynor et al. Supplemental Figure 1

## Slide 2
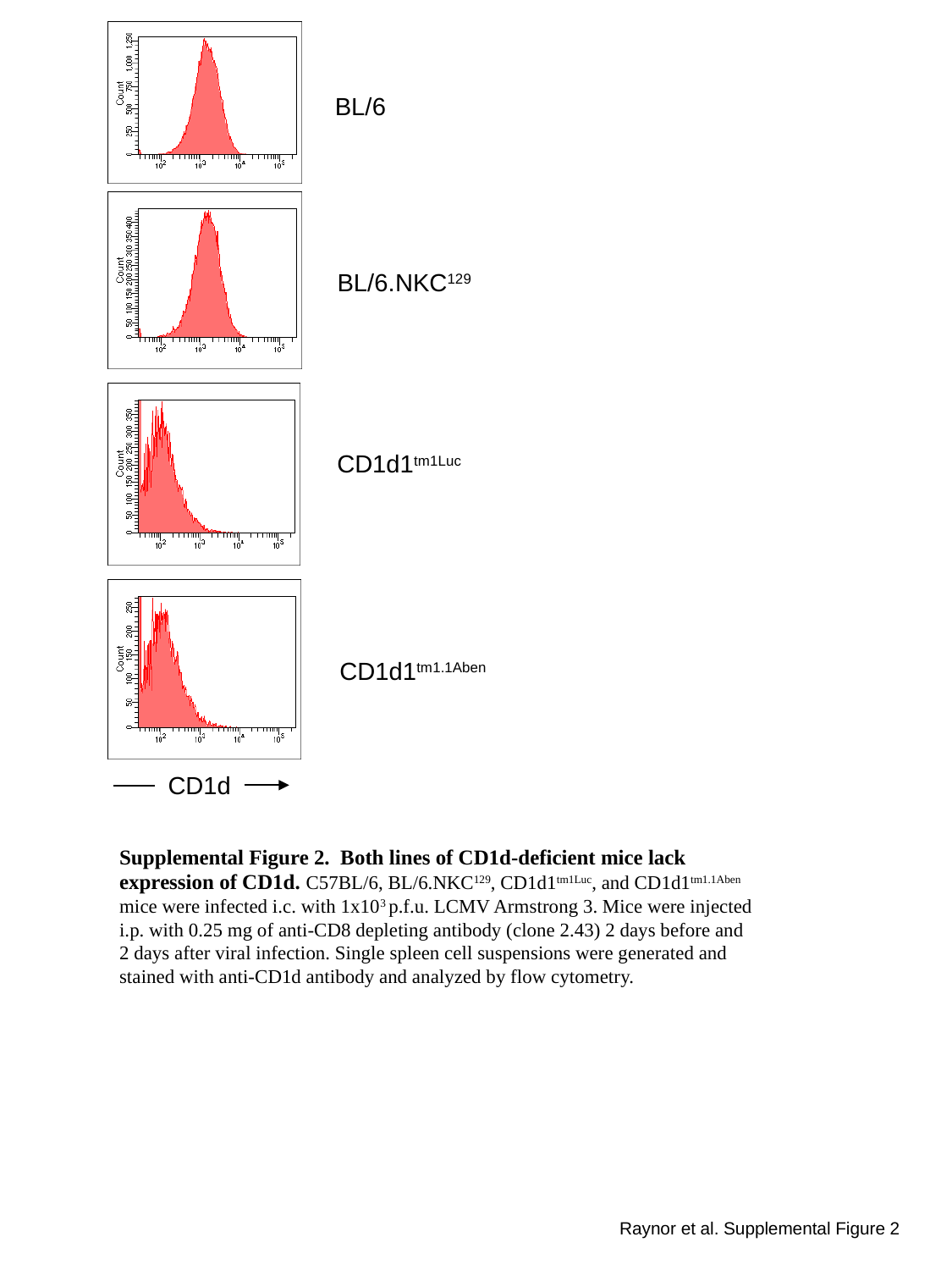

BL/6
BL/6.NKC129
CD1d1tm1Luc
CD1d1tm1.1Aben
CD1d
Supplemental Figure 2. Both lines of CD1d-deficient mice lack expression of CD1d. C57BL/6, BL/6.NKC129, CD1d1tm1Luc, and CD1d1tm1.1Aben mice were infected i.c. with 1x10^3^ p.f.u. LCMV Armstrong 3. Mice were injected i.p. with 0.25 mg of anti-CD8 depleting antibody (clone 2.43) 2 days before and 2 days after viral infection. Single spleen cell suspensions were generated and stained with anti-CD1d antibody and analyzed by flow cytometry.
Raynor et al. Supplemental Figure 2

## Slide 3
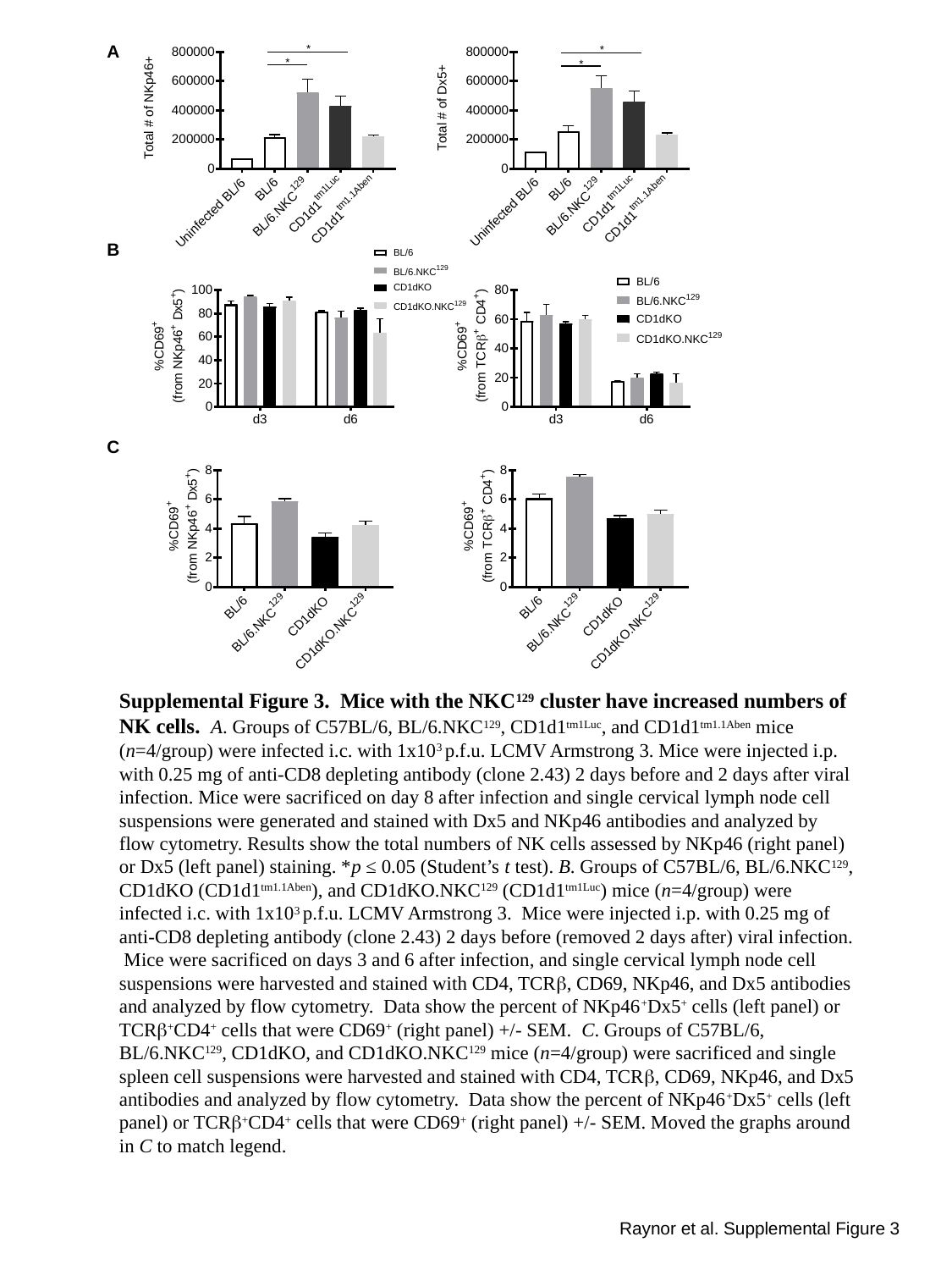

A
B
C
Supplemental Figure 3. Mice with the NKC129 cluster have increased numbers of NK cells. A. Groups of C57BL/6, BL/6.NKC129, CD1d1tm1Luc, and CD1d1tm1.1Aben mice (n=4/group) were infected i.c. with 1x10^3^ p.f.u. LCMV Armstrong 3. Mice were injected i.p. with 0.25 mg of anti-CD8 depleting antibody (clone 2.43) 2 days before and 2 days after viral infection. Mice were sacrificed on day 8 after infection and single cervical lymph node cell suspensions were generated and stained with Dx5 and NKp46 antibodies and analyzed by flow cytometry. Results show the total numbers of NK cells assessed by NKp46 (right panel) or Dx5 (left panel) staining. *p ≤ 0.05 (Student’s t test). B. Groups of C57BL/6, BL/6.NKC129, CD1dKO (CD1d1tm1.1Aben), and CD1dKO.NKC129 (CD1d1tm1Luc) mice (n=4/group) were infected i.c. with 1x10^3^ p.f.u. LCMV Armstrong 3. Mice were injected i.p. with 0.25 mg of anti-CD8 depleting antibody (clone 2.43) 2 days before (removed 2 days after) viral infection. Mice were sacrificed on days 3 and 6 after infection, and single cervical lymph node cell suspensions were harvested and stained with CD4, TCRb, CD69, NKp46, and Dx5 antibodies and analyzed by flow cytometry. Data show the percent of NKp46+Dx5+ cells (left panel) or TCRb+CD4+ cells that were CD69+ (right panel) +/- SEM. C. Groups of C57BL/6, BL/6.NKC129, CD1dKO, and CD1dKO.NKC129 mice (n=4/group) were sacrificed and single spleen cell suspensions were harvested and stained with CD4, TCRb, CD69, NKp46, and Dx5 antibodies and analyzed by flow cytometry. Data show the percent of NKp46+Dx5+ cells (left panel) or TCRb+CD4+ cells that were CD69+ (right panel) +/- SEM. Moved the graphs around in C to match legend.
Raynor et al. Supplemental Figure 3

## Slide 4
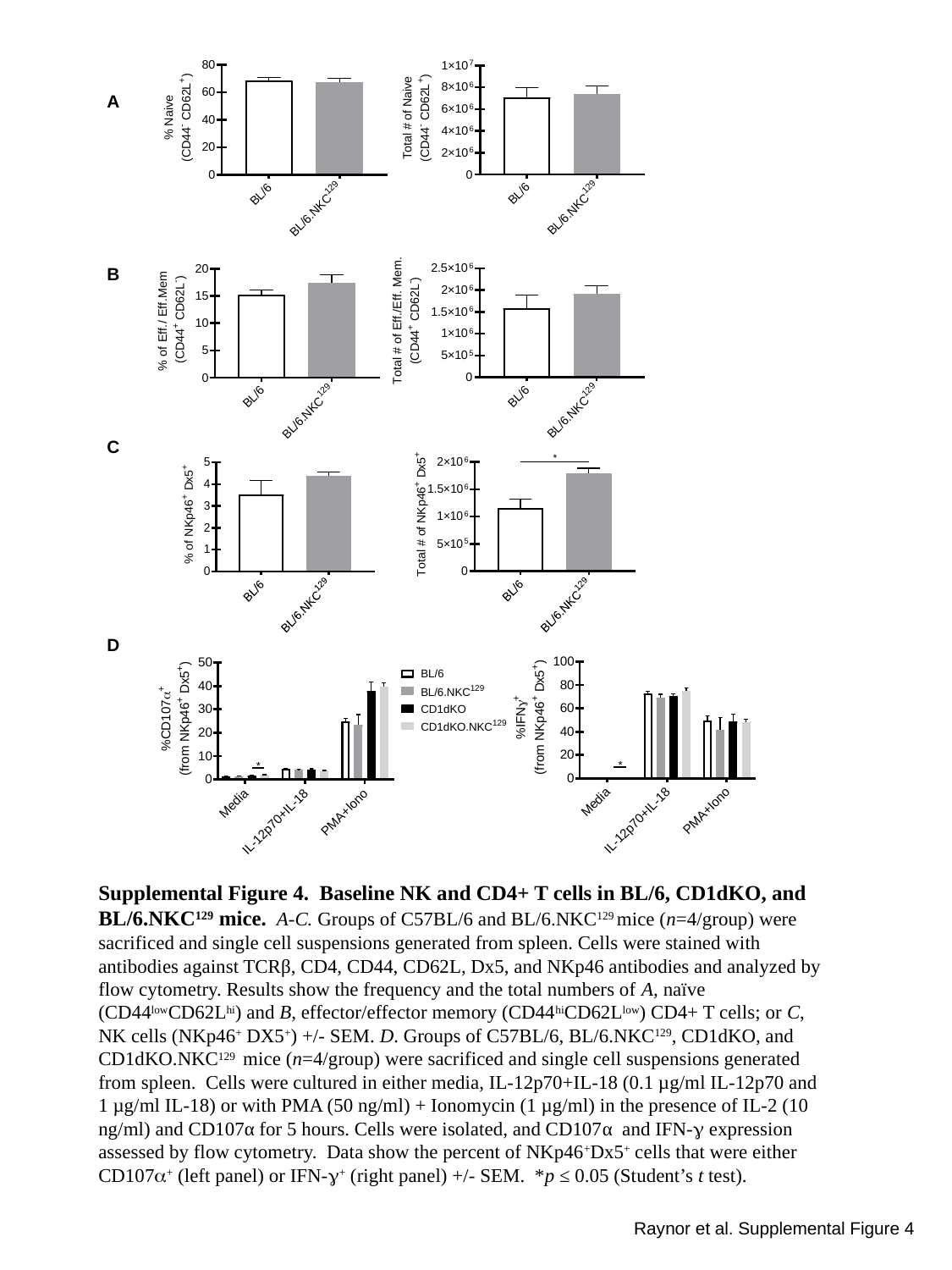

A
B
C
D
Supplemental Figure 4. Baseline NK and CD4+ T cells in BL/6, CD1dKO, and BL/6.NKC129 mice. A-C. Groups of C57BL/6 and BL/6.NKC129 mice (n=4/group) were sacrificed and single cell suspensions generated from spleen. Cells were stained with antibodies against TCRβ, CD4, CD44, CD62L, Dx5, and NKp46 antibodies and analyzed by flow cytometry. Results show the frequency and the total numbers of A, naïve (CD44lowCD62Lhi) and B, effector/effector memory (CD44hiCD62Llow) CD4+ T cells; or C, NK cells (NKp46+ DX5+) +/- SEM. D. Groups of C57BL/6, BL/6.NKC129, CD1dKO, and CD1dKO.NKC129 mice (n=4/group) were sacrificed and single cell suspensions generated from spleen. Cells were cultured in either media, IL-12p70+IL-18 (0.1 µg/ml IL-12p70 and 1 µg/ml IL-18) or with PMA (50 ng/ml) + Ionomycin (1 µg/ml) in the presence of IL-2 (10 ng/ml) and CD107α for 5 hours. Cells were isolated, and CD107α and IFN-g expression assessed by flow cytometry. Data show the percent of NKp46+Dx5+ cells that were either CD107a+ (left panel) or IFN-g+ (right panel) +/- SEM. *p ≤ 0.05 (Student’s t test).
Raynor et al. Supplemental Figure 4

## Slide 5
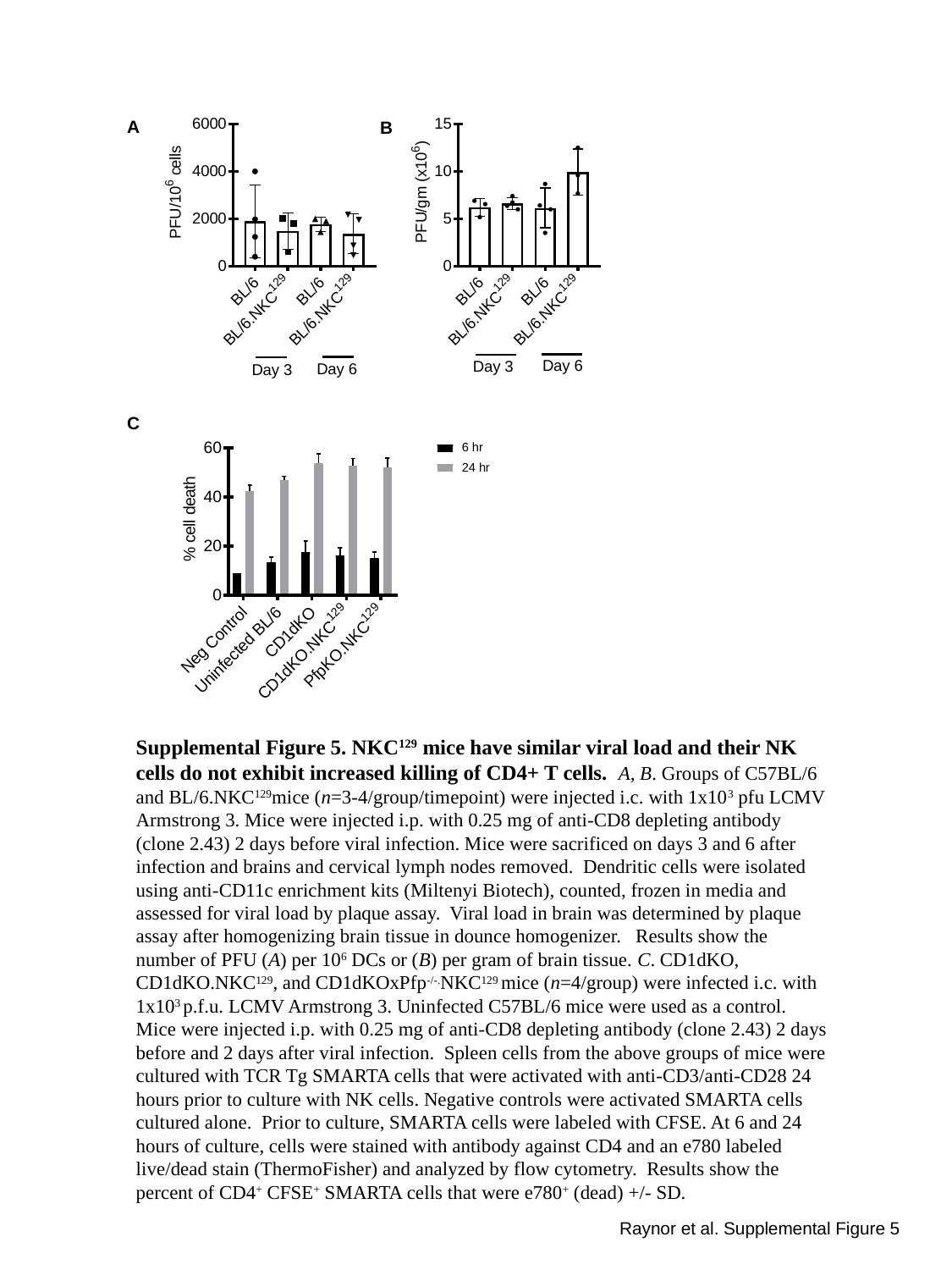

A
C
B
Supplemental Figure 5. NKC129 mice have similar viral load and their NK cells do not exhibit increased killing of CD4+ T cells. A, B. Groups of C57BL/6 and BL/6.NKC129mice (n=3-4/group/timepoint) were injected i.c. with 1x10^3^ pfu LCMV Armstrong 3. Mice were injected i.p. with 0.25 mg of anti-CD8 depleting antibody (clone 2.43) 2 days before viral infection. Mice were sacrificed on days 3 and 6 after infection and brains and cervical lymph nodes removed. Dendritic cells were isolated using anti-CD11c enrichment kits (Miltenyi Biotech), counted, frozen in media and assessed for viral load by plaque assay. Viral load in brain was determined by plaque assay after homogenizing brain tissue in dounce homogenizer. Results show the number of PFU (A) per 106 DCs or (B) per gram of brain tissue. C. CD1dKO, CD1dKO.NKC129, and CD1dKOxPfp-/-.NKC129 mice (n=4/group) were infected i.c. with 1x10^3^ p.f.u. LCMV Armstrong 3. Uninfected C57BL/6 mice were used as a control. Mice were injected i.p. with 0.25 mg of anti-CD8 depleting antibody (clone 2.43) 2 days before and 2 days after viral infection. Spleen cells from the above groups of mice were cultured with TCR Tg SMARTA cells that were activated with anti-CD3/anti-CD28 24 hours prior to culture with NK cells. Negative controls were activated SMARTA cells cultured alone. Prior to culture, SMARTA cells were labeled with CFSE. At 6 and 24 hours of culture, cells were stained with antibody against CD4 and an e780 labeled live/dead stain (ThermoFisher) and analyzed by flow cytometry. Results show the percent of CD4+ CFSE+ SMARTA cells that were e780+ (dead) +/- SD.
Raynor et al. Supplemental Figure 5

## Slide 6
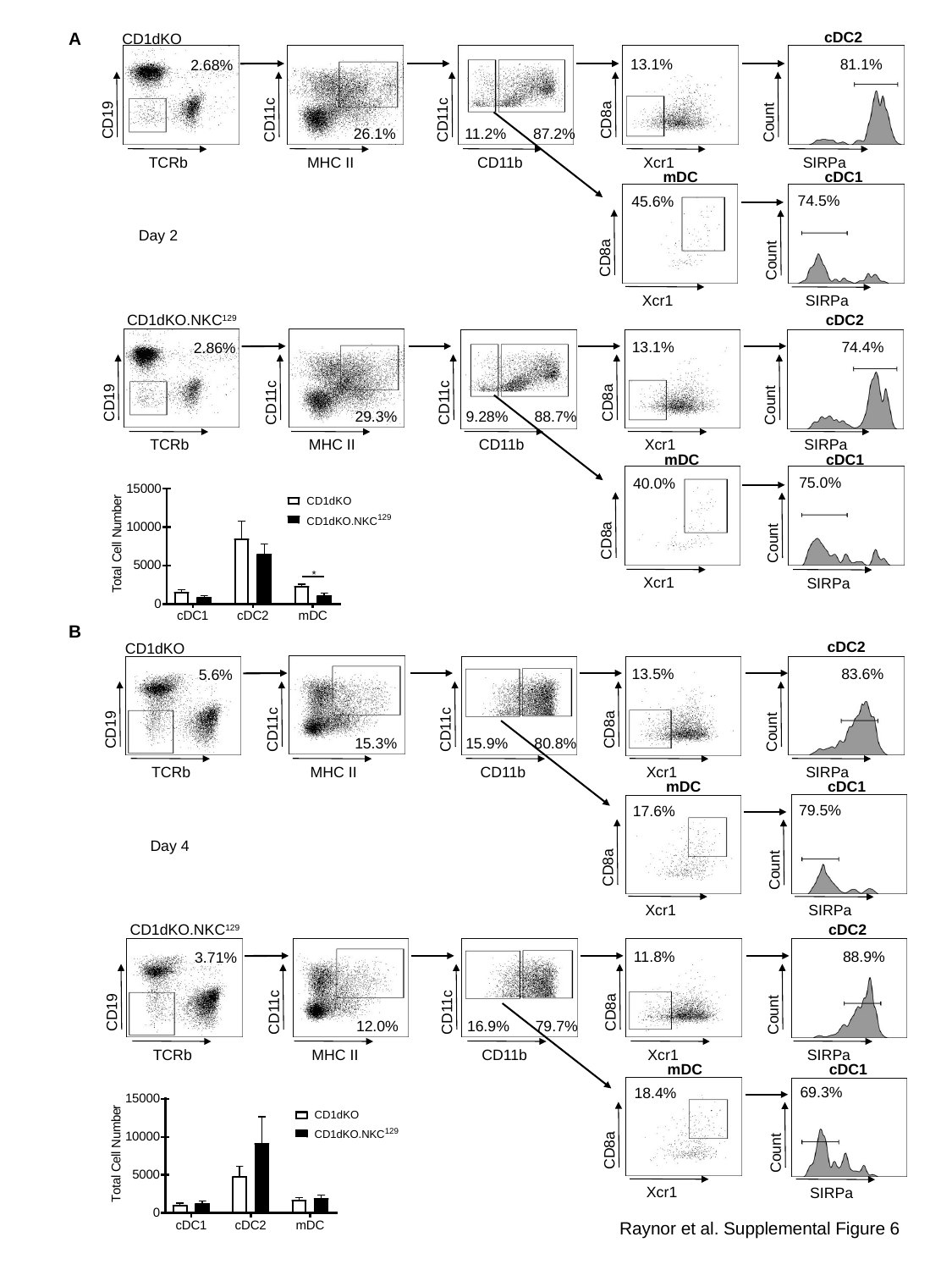

A
B
cDC2
13.1%
81.1%
2.68%
CD19
TCRb
CD11c
MHC II
CD11c
CD11b
CD8a
Xcr1
Count
SIRPa
26.1%
11.2%
87.2%
mDC
cDC1
74.5%
45.6%
CD8a
Xcr1
Count
SIRPa
CD1dKO
cDC2
13.1%
74.4%
2.86%
CD19
TCRb
CD11c
MHC II
CD11c
CD11b
CD8a
Xcr1
Count
SIRPa
29.3%
9.28%
88.7%
mDC
cDC1
75.0%
40.0%
CD8a
Xcr1
Count
SIRPa
CD1dKO.NKC129
Day 2
cDC2
13.5%
83.6%
5.6%
CD19
TCRb
CD11c
MHC II
CD11c
CD11b
CD8a
Xcr1
Count
SIRPa
15.3%
15.9%
80.8%
mDC
cDC1
79.5%
17.6%
CD8a
Xcr1
Count
SIRPa
CD1dKO
cDC2
11.8%
88.9%
3.71%
CD19
TCRb
CD11c
MHC II
CD11c
CD11b
CD8a
Xcr1
Count
SIRPa
12.0%
16.9%
79.7%
mDC
cDC1
69.3%
18.4%
CD8a
Xcr1
Count
SIRPa
CD1dKO.NKC129
Day 4
Raynor et al. Supplemental Figure 6

## Slide 7
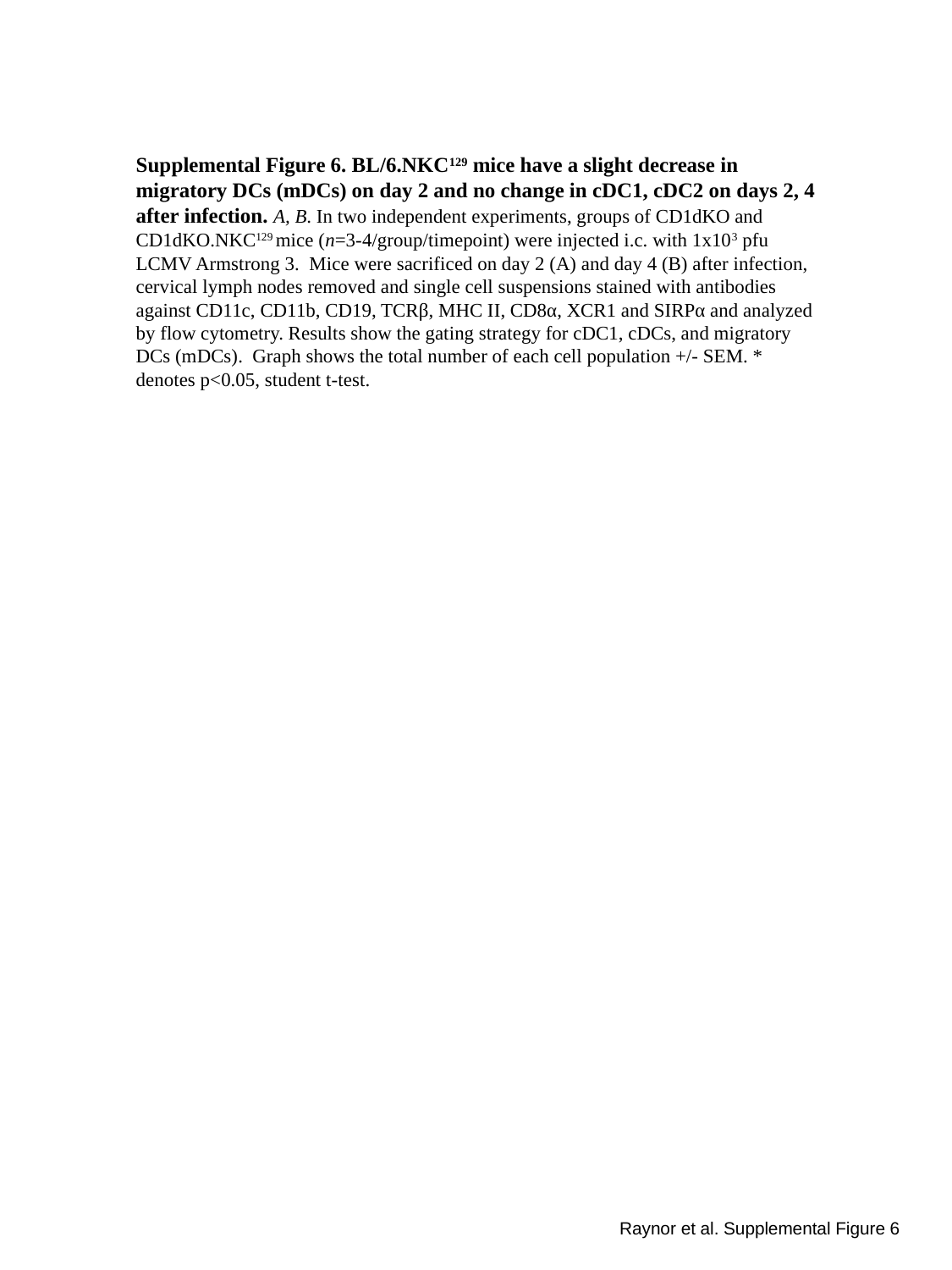

Supplemental Figure 6. BL/6.NKC129 mice have a slight decrease in migratory DCs (mDCs) on day 2 and no change in cDC1, cDC2 on days 2, 4 after infection. A, B. In two independent experiments, groups of CD1dKO and CD1dKO.NKC129 mice (n=3-4/group/timepoint) were injected i.c. with 1x10^3^ pfu LCMV Armstrong 3. Mice were sacrificed on day 2 (A) and day 4 (B) after infection, cervical lymph nodes removed and single cell suspensions stained with antibodies against CD11c, CD11b, CD19, TCRβ, MHC II, CD8α, XCR1 and SIRPα and analyzed by flow cytometry. Results show the gating strategy for cDC1, cDCs, and migratory DCs (mDCs). Graph shows the total number of each cell population +/- SEM. * denotes p<0.05, student t-test.
Raynor et al. Supplemental Figure 6

## Slide 8
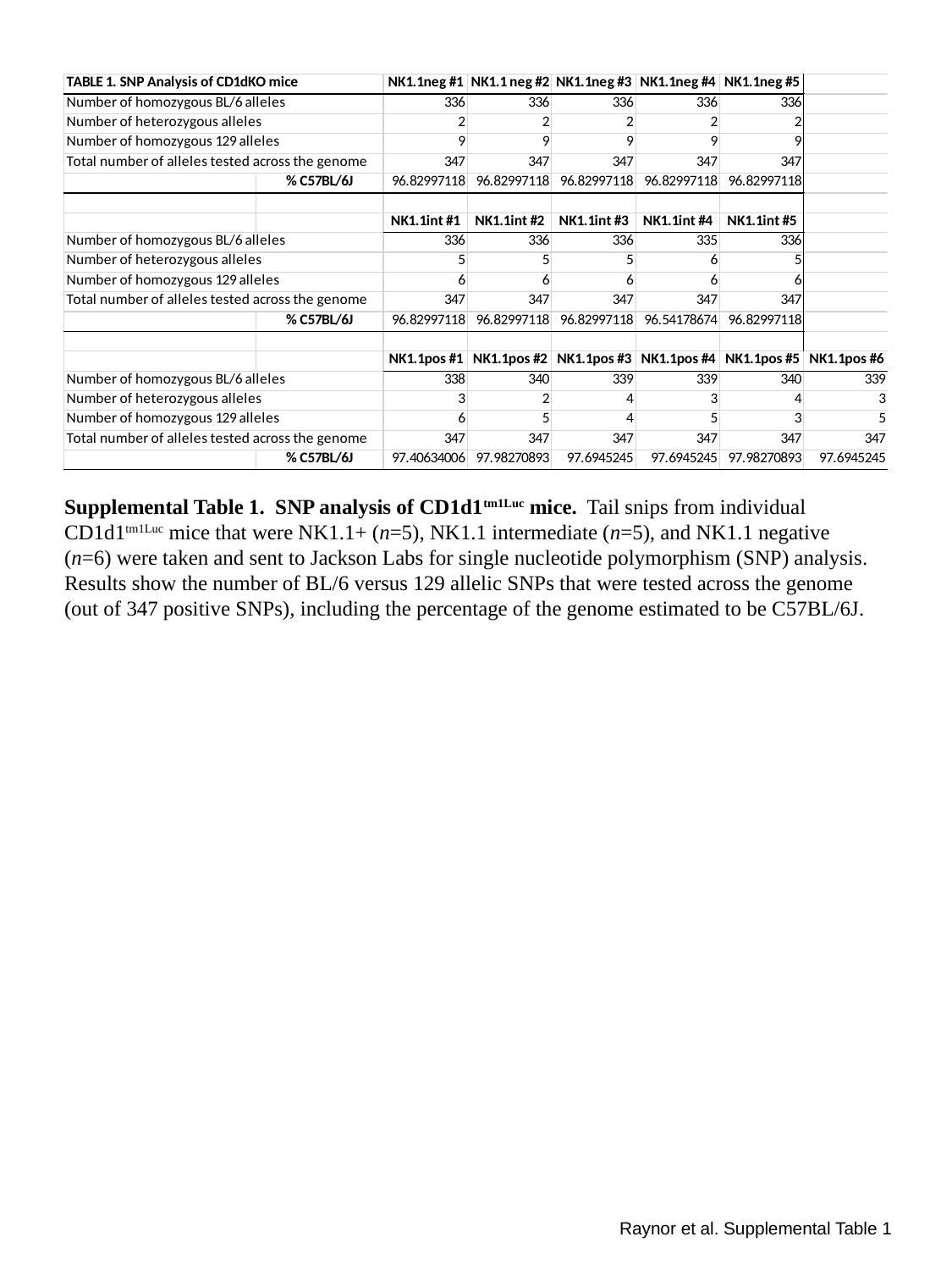

Supplemental Table 1. SNP analysis of CD1d1tm1Luc mice. Tail snips from individual CD1d1tm1Luc mice that were NK1.1+ (n=5), NK1.1 intermediate (n=5), and NK1.1 negative (n=6) were taken and sent to Jackson Labs for single nucleotide polymorphism (SNP) analysis. Results show the number of BL/6 versus 129 allelic SNPs that were tested across the genome (out of 347 positive SNPs), including the percentage of the genome estimated to be C57BL/6J.
Raynor et al. Supplemental Table 1

## Slide 9
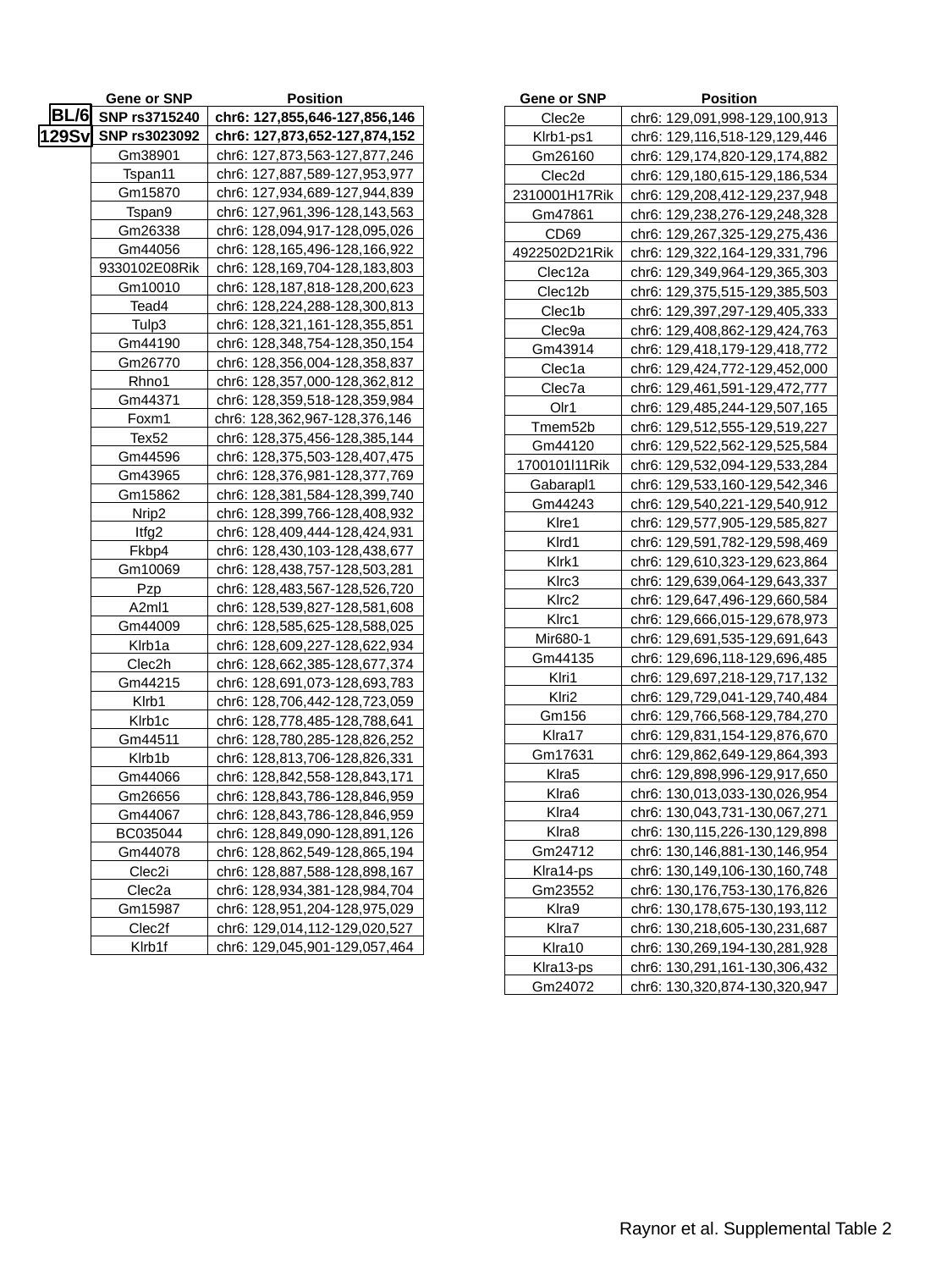

| Gene or SNP | Position |
| --- | --- |
| Clec2e | chr6: 129,091,998-129,100,913 |
| Klrb1-ps1 | chr6: 129,116,518-129,129,446 |
| Gm26160 | chr6: 129,174,820-129,174,882 |
| Clec2d | chr6: 129,180,615-129,186,534 |
| 2310001H17Rik | chr6: 129,208,412-129,237,948 |
| Gm47861 | chr6: 129,238,276-129,248,328 |
| CD69 | chr6: 129,267,325-129,275,436 |
| 4922502D21Rik | chr6: 129,322,164-129,331,796 |
| Clec12a | chr6: 129,349,964-129,365,303 |
| Clec12b | chr6: 129,375,515-129,385,503 |
| Clec1b | chr6: 129,397,297-129,405,333 |
| Clec9a | chr6: 129,408,862-129,424,763 |
| Gm43914 | chr6: 129,418,179-129,418,772 |
| Clec1a | chr6: 129,424,772-129,452,000 |
| Clec7a | chr6: 129,461,591-129,472,777 |
| Olr1 | chr6: 129,485,244-129,507,165 |
| Tmem52b | chr6: 129,512,555-129,519,227 |
| Gm44120 | chr6: 129,522,562-129,525,584 |
| 1700101l11Rik | chr6: 129,532,094-129,533,284 |
| Gabarapl1 | chr6: 129,533,160-129,542,346 |
| Gm44243 | chr6: 129,540,221-129,540,912 |
| Klre1 | chr6: 129,577,905-129,585,827 |
| Klrd1 | chr6: 129,591,782-129,598,469 |
| Klrk1 | chr6: 129,610,323-129,623,864 |
| Klrc3 | chr6: 129,639,064-129,643,337 |
| Klrc2 | chr6: 129,647,496-129,660,584 |
| Klrc1 | chr6: 129,666,015-129,678,973 |
| Mir680-1 | chr6: 129,691,535-129,691,643 |
| Gm44135 | chr6: 129,696,118-129,696,485 |
| Klri1 | chr6: 129,697,218-129,717,132 |
| Klri2 | chr6: 129,729,041-129,740,484 |
| Gm156 | chr6: 129,766,568-129,784,270 |
| Klra17 | chr6: 129,831,154-129,876,670 |
| Gm17631 | chr6: 129,862,649-129,864,393 |
| Klra5 | chr6: 129,898,996-129,917,650 |
| Klra6 | chr6: 130,013,033-130,026,954 |
| Klra4 | chr6: 130,043,731-130,067,271 |
| Klra8 | chr6: 130,115,226-130,129,898 |
| Gm24712 | chr6: 130,146,881-130,146,954 |
| Klra14-ps | chr6: 130,149,106-130,160,748 |
| Gm23552 | chr6: 130,176,753-130,176,826 |
| Klra9 | chr6: 130,178,675-130,193,112 |
| Klra7 | chr6: 130,218,605-130,231,687 |
| Klra10 | chr6: 130,269,194-130,281,928 |
| Klra13-ps | chr6: 130,291,161-130,306,432 |
| Gm24072 | chr6: 130,320,874-130,320,947 |
| Gene or SNP | Position |
| --- | --- |
| SNP rs3715240 | chr6: 127,855,646-127,856,146 |
| SNP rs3023092 | chr6: 127,873,652-127,874,152 |
| Gm38901 | chr6: 127,873,563-127,877,246 |
| Tspan11 | chr6: 127,887,589-127,953,977 |
| Gm15870 | chr6: 127,934,689-127,944,839 |
| Tspan9 | chr6: 127,961,396-128,143,563 |
| Gm26338 | chr6: 128,094,917-128,095,026 |
| Gm44056 | chr6: 128,165,496-128,166,922 |
| 9330102E08Rik | chr6: 128,169,704-128,183,803 |
| Gm10010 | chr6: 128,187,818-128,200,623 |
| Tead4 | chr6: 128,224,288-128,300,813 |
| Tulp3 | chr6: 128,321,161-128,355,851 |
| Gm44190 | chr6: 128,348,754-128,350,154 |
| Gm26770 | chr6: 128,356,004-128,358,837 |
| Rhno1 | chr6: 128,357,000-128,362,812 |
| Gm44371 | chr6: 128,359,518-128,359,984 |
| Foxm1 | chr6: 128,362,967-128,376,146 |
| Tex52 | chr6: 128,375,456-128,385,144 |
| Gm44596 | chr6: 128,375,503-128,407,475 |
| Gm43965 | chr6: 128,376,981-128,377,769 |
| Gm15862 | chr6: 128,381,584-128,399,740 |
| Nrip2 | chr6: 128,399,766-128,408,932 |
| Itfg2 | chr6: 128,409,444-128,424,931 |
| Fkbp4 | chr6: 128,430,103-128,438,677 |
| Gm10069 | chr6: 128,438,757-128,503,281 |
| Pzp | chr6: 128,483,567-128,526,720 |
| A2ml1 | chr6: 128,539,827-128,581,608 |
| Gm44009 | chr6: 128,585,625-128,588,025 |
| Klrb1a | chr6: 128,609,227-128,622,934 |
| Clec2h | chr6: 128,662,385-128,677,374 |
| Gm44215 | chr6: 128,691,073-128,693,783 |
| Klrb1 | chr6: 128,706,442-128,723,059 |
| Klrb1c | chr6: 128,778,485-128,788,641 |
| Gm44511 | chr6: 128,780,285-128,826,252 |
| Klrb1b | chr6: 128,813,706-128,826,331 |
| Gm44066 | chr6: 128,842,558-128,843,171 |
| Gm26656 | chr6: 128,843,786-128,846,959 |
| Gm44067 | chr6: 128,843,786-128,846,959 |
| BC035044 | chr6: 128,849,090-128,891,126 |
| Gm44078 | chr6: 128,862,549-128,865,194 |
| Clec2i | chr6: 128,887,588-128,898,167 |
| Clec2a | chr6: 128,934,381-128,984,704 |
| Gm15987 | chr6: 128,951,204-128,975,029 |
| Clec2f | chr6: 129,014,112-129,020,527 |
| Klrb1f | chr6: 129,045,901-129,057,464 |
BL/6
129Sv
Raynor et al. Supplemental Table 2

## Slide 10
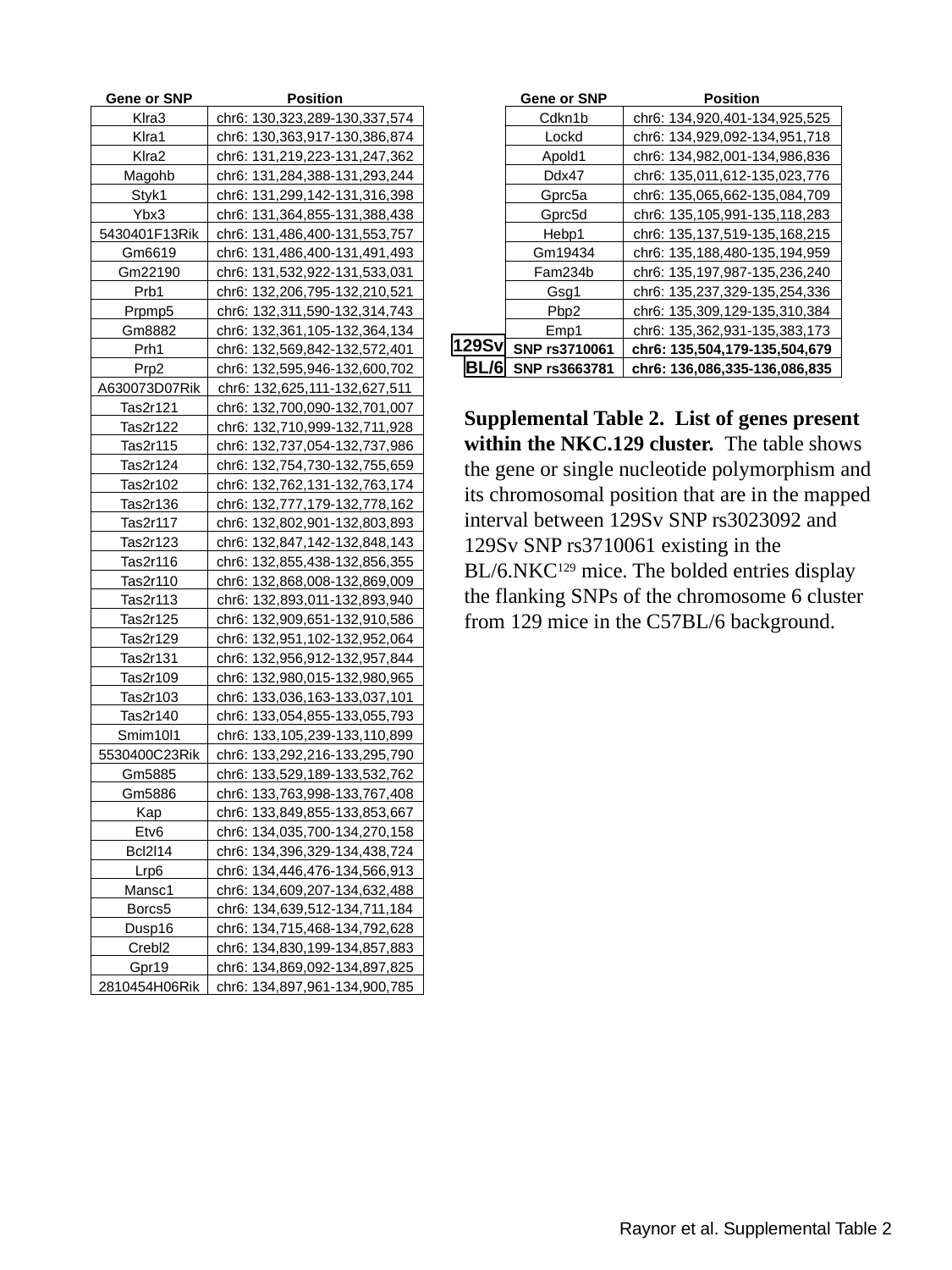

| Gene or SNP | Position |
| --- | --- |
| Klra3 | chr6: 130,323,289-130,337,574 |
| Klra1 | chr6: 130,363,917-130,386,874 |
| Klra2 | chr6: 131,219,223-131,247,362 |
| Magohb | chr6: 131,284,388-131,293,244 |
| Styk1 | chr6: 131,299,142-131,316,398 |
| Ybx3 | chr6: 131,364,855-131,388,438 |
| 5430401F13Rik | chr6: 131,486,400-131,553,757 |
| Gm6619 | chr6: 131,486,400-131,491,493 |
| Gm22190 | chr6: 131,532,922-131,533,031 |
| Prb1 | chr6: 132,206,795-132,210,521 |
| Prpmp5 | chr6: 132,311,590-132,314,743 |
| Gm8882 | chr6: 132,361,105-132,364,134 |
| Prh1 | chr6: 132,569,842-132,572,401 |
| Prp2 | chr6: 132,595,946-132,600,702 |
| A630073D07Rik | chr6: 132,625,111-132,627,511 |
| Tas2r121 | chr6: 132,700,090-132,701,007 |
| Tas2r122 | chr6: 132,710,999-132,711,928 |
| Tas2r115 | chr6: 132,737,054-132,737,986 |
| Tas2r124 | chr6: 132,754,730-132,755,659 |
| Tas2r102 | chr6: 132,762,131-132,763,174 |
| Tas2r136 | chr6: 132,777,179-132,778,162 |
| Tas2r117 | chr6: 132,802,901-132,803,893 |
| Tas2r123 | chr6: 132,847,142-132,848,143 |
| Tas2r116 | chr6: 132,855,438-132,856,355 |
| Tas2r110 | chr6: 132,868,008-132,869,009 |
| Tas2r113 | chr6: 132,893,011-132,893,940 |
| Tas2r125 | chr6: 132,909,651-132,910,586 |
| Tas2r129 | chr6: 132,951,102-132,952,064 |
| Tas2r131 | chr6: 132,956,912-132,957,844 |
| Tas2r109 | chr6: 132,980,015-132,980,965 |
| Tas2r103 | chr6: 133,036,163-133,037,101 |
| Tas2r140 | chr6: 133,054,855-133,055,793 |
| Smim10l1 | chr6: 133,105,239-133,110,899 |
| 5530400C23Rik | chr6: 133,292,216-133,295,790 |
| Gm5885 | chr6: 133,529,189-133,532,762 |
| Gm5886 | chr6: 133,763,998-133,767,408 |
| Kap | chr6: 133,849,855-133,853,667 |
| Etv6 | chr6: 134,035,700-134,270,158 |
| Bcl2l14 | chr6: 134,396,329-134,438,724 |
| Lrp6 | chr6: 134,446,476-134,566,913 |
| Mansc1 | chr6: 134,609,207-134,632,488 |
| Borcs5 | chr6: 134,639,512-134,711,184 |
| Dusp16 | chr6: 134,715,468-134,792,628 |
| Crebl2 | chr6: 134,830,199-134,857,883 |
| Gpr19 | chr6: 134,869,092-134,897,825 |
| 2810454H06Rik | chr6: 134,897,961-134,900,785 |
| Gene or SNP | Position |
| --- | --- |
| Cdkn1b | chr6: 134,920,401-134,925,525 |
| Lockd | chr6: 134,929,092-134,951,718 |
| Apold1 | chr6: 134,982,001-134,986,836 |
| Ddx47 | chr6: 135,011,612-135,023,776 |
| Gprc5a | chr6: 135,065,662-135,084,709 |
| Gprc5d | chr6: 135,105,991-135,118,283 |
| Hebp1 | chr6: 135,137,519-135,168,215 |
| Gm19434 | chr6: 135,188,480-135,194,959 |
| Fam234b | chr6: 135,197,987-135,236,240 |
| Gsg1 | chr6: 135,237,329-135,254,336 |
| Pbp2 | chr6: 135,309,129-135,310,384 |
| Emp1 | chr6: 135,362,931-135,383,173 |
| SNP rs3710061 | chr6: 135,504,179-135,504,679 |
| SNP rs3663781 | chr6: 136,086,335-136,086,835 |
129Sv
BL/6
Supplemental Table 2. List of genes present within the NKC.129 cluster. The table shows the gene or single nucleotide polymorphism and its chromosomal position that are in the mapped interval between 129Sv SNP rs3023092 and 129Sv SNP rs3710061 existing in the BL/6.NKC129 mice. The bolded entries display the flanking SNPs of the chromosome 6 cluster from 129 mice in the C57BL/6 background.
Raynor et al. Supplemental Table 2
